# Effect of Genetic Variation in CYP450 on Gonadal Impairment in a European Cohort of Female Childhood Cancer Survivors, Based on a Candidate Gene Approach: Results from the PanCareLIFE Study

**DOI:** 10.3390/cancers13184598

**Published:** 2021-09-13

**Authors:** M. E. Madeleine van der Perk, Linda Broer, Yutaka Yasui, Leslie L. Robison, Melissa M. Hudson, Joop S. E. Laven, Helena J. van der Pal, Wim J. E. Tissing, Birgitta Versluys, Dorine Bresters, Gertjan J. L. Kaspers, Andrica C. H. de Vries, Cornelis B. Lambalk, Annelies Overbeek, Jacqueline J. Loonen, Catharina C. M. Beerendonk, Julianne Byrne, Claire Berger, Eva Clemens, Uta Dirksen, Jeanette Falck Winther, Sophie D. Fosså, Desiree Grabow, Monica Muraca, Melanie Kaiser, Tomáš Kepák, Jarmila Kruseova, Dalit Modan-Moses, Claudia Spix, Oliver Zolk, Peter Kaatsch, Jesse H. Krijthe, Leontien C. M. Kremer, Russell J. Brooke, Jessica L. Baedke, Ron H. N. van Schaik, John N. van den Anker, André G. Uitterlinden, Annelies M. E. Bos, Flora E. van Leeuwen, Eline van Dulmen-den Broeder, Anne-Lotte L. F. van der Kooi, Marry M. van den Heuvel-Eibrink, on behalf of the PanCareLIFE Consortium

**Affiliations:** 1 Princess Máxima Center for Pediatric Oncology, 3584 CS Utrecht, The Netherlands; H.J.H.vanderPal@prinsesmaximacentrum.nl (H.J.v.d.P.); w.j.e.tissing@prinsesmaximacentrum.nl (W.J.E.T.); A.B.Versluijs@prinsesmaximacentrum.nl (B.V.); D.Bresters@prinsesmaximacentrum.nl (D.B.); g.j.l.kaspers@prinsesmaximacentrum.nl (G.J.L.K.); A.C.H.deVries-15@prinsesmaximacentrum.nl (A.C.H.d.V.); clemens.eva@gmail.com (E.C.); l.c.m.kremer@prinsesmaximacentrum.nl (L.C.M.K.); e.vandulmen-denbroeder@amsterdamumc.nl (E.v.D.-d.B.); a.vanderkooi@erasmusmc.nl (A.-L.L.F.v.d.K.); m.m.vandenheuvel-eibrink@prinsesmaximacentrum.nl (M.M.v.d.H.-E.); 2 Department of Internal Medicine, Rotterdam, ErasmusMC University Medical Center Rotterdam, 3015 GD Rotterdam, The Netherlands; l.broer@erasmusmc.nl (L.B.); a.g.uitterlinden@erasmusmc.nl (A.G.U.); 3 Department of Epidemiology and Cancer Control, St. Jude Children’s Research Hospital, Memphis, TN 38105, USA; yutaka.yasui@stjude.org (Y.Y.); les.robison@stjude.org (L.L.R.); melissa.hudson@stjude.org (M.M.H.); johnbrooke@gmail.com (R.J.B.); jessica.baedke@stjude.org (J.L.B.); 4 Department of Oncology, Division of Survivorship, St. Jude Children’s Research Hospital, Memphis, TN 38105, USA; 5 Department of Obstetrics and Gynecology, Erasmus MC–University Medical Center, 3015 GD Rotterdam, The Netherlands; j.laven@erasmusmc.nl; 6 Department of Pediatric Oncology-Haematology, Emma Children’s Hospital, Amsterdam UMC, Vrije Universiteit Amsterdam, 1105 AZ Amsterdam, The Netherlands; 7 Department of Obstetrics and Gynaecology, Amsterdam UMC, Vrije Universiteit Amsterdam, 1105 AZ Amsterdam, The Netherlands; cb.lambalk@amsterdamumc.nl (C.B.L.); a.overbeek@nwz.nl (A.O.); 8 Department of Haematology, Radboud University Medical Center, 6500 HB Nijmegen, The Netherlands; Jacqueline.Loonen@radboudumc.nl; 9 Department of Obstetrics and Gynaecology, Radboud University Medical Center, 6500 HB Nijmegen, The Netherlands; Ina.Beerendonk@radboudumc.nl; 10 Boyne Research Institute, 5 Bolton Square, East, Drogheda, A92 RY6K Co. Louth, Ireland; jbyrne@boyneresearch.ie; 11 Department of Paediatric Oncology, University Hospital, 42 055 Saint-Etienne, France; claire.berger@chu-st-etienne.fr; 12 Lyon University, Jean Monnet University, INSERM, U 1059, Sainbiose, 42023 Saint-Etienne, France; 13 University Hospital Essen, Pediatrics III, West German Cancer Centre, 45147 Essen, Germany; Uta.Dirksen@uk-essen.de; 14 German Cancer Research Centre, DKTK, Site Essen, 45147 Essen, Germany; 15 Childhood Cancer Research Group, Danish Cancer Society Research Center, 2100 Copenhagen, Denmark; jeanette@cancer.dk; 16 Department of Clinical Medicine, Faculty of Health, Aarhus University and University Hospital, 8200 Aarhus, Denmark; 17 Department of Oncology, Oslo University Hospital, 0372 Oslo, Norway; sopfos@ous-hf.no; 18 Division of Childhood Cancer Epidemiology, German Childhood Cancer Registry, Institute of Medical Biostatistics, Epidemiology and Informatics (IMBEI), University Medical Center of the Johannes Gutenberg University Mainz, 55131 Mainz, Germany; desiree.grabow@uni-mainz.de (D.G.); kaiserm@uni-mainz.de (M.K.); clauspix@uni-mainz.de (C.S.); kaatsch@uni-mainz.de (P.K.); 19 Epidemiology and Biostatistics Unit and DOPO Clinic, IRCCS Istituto Giannina Gaslini, 16147 Genova, Italy; monicamuraca@gaslini.org; 20 University Hospital Brno, International Clinical Research Center (FNUSA-ICRC), Masaryk University, 656 91 Brno, Czech Republic; Kepak.Tomas@fnbrno.cz; 21 Motol University Hospital, 150 05 Prague, Czech Republic; jarmila.Kruseova@fnmotol.cz; 22 The Edmond and Lily Safra Children’s Hospital, Chaim Sheba Medical Center, Tel Hashomer, and the Sackler Faculty of Medicine, Tel-Aviv University, Tel-Aviv 6997801, Israel; dalit.modan@sheba.health.gov.il; 23 Institute of Clinical Pharmacology, Brandenburg Medical School Theodor Fontane, Immanuel Klinik Rüdersdorf, 16816 Neuruppin, Germany; oliver.zolk@mhb-fontane.de; 24 Department of Intelligent Systems, Delft University of Technology, 2628 BL Delft, The Netherlands; jkrijthe@gmail.com; 25 Department of clinical chemistry, Erasmus MC University Medical Center Rotterdam, 3015 GD Rotterdam, The Netherlands; r.vanschaik@erasmusmc.nl; 26 Division of Clinical Pharmacology, Children’s National Hospital, Washington, DC 20010, USA; jvandena@childrensnational.org; 27 Department of Reproductive Medicine and Gynecology, University Medical Center Utrecht, 3584 CS Utrecht, The Netherlands; A.Bos-8@umcutrecht.nl; 28 Department of Epidemiology, Netherlands Cancer Institute, 1066 CX Amsterdam, The Netherlands; f.v.leeuwen@nki.nl

**Keywords:** childhood cancer survivors, ovarian function, anti-Müllerian hormone, chemotherapy, candidate gene approach, cytochrome P450 genes

## Abstract

**Simple Summary:**

Childhood cancer patients receiving treatment containing alkylating agents are at risk of infertility, yet inter-individual variability in treatment-related ovarian damage is observed. Alkylating agents are metabolized by cytochrome P450 (CYP450) enzymes and polymorphisms in these CYP450 enzymes may explain this variability in ovarian damage. This study on genetic variation in CYP450 enzymes of chemotherapy-induced gonadotoxicity, using anti-Müllerian hormone (AMH) levels as a proxy for ovarian reserve, in female childhood cancer survivors (CCSs) may identify patients at risk of infertility. This unique global collaboration of two large CCS studies shows the significant gonadotoxic effect of enzyme CYP3A4*3 and significant protective effect of CYP2B6*2 on gonadal function in CCSs receiving alkylating agents. Genetic variation in CYP3A4 and CYP2B6 have previously been associated with gonadotoxicity after cancer treatment. These findings could guide risk prediction models determining patients at risk of chemotherapy-induced gonadal impairment.

**Abstract:**

Background: Female childhood cancer survivors (CCSs) carry a risk of therapy-related gonadal dysfunction. Alkylating agents (AA) are well-established risk factors, yet inter-individual variability in ovarian function is observed. Polymorphisms in CYP450 enzymes may explain this variability in AA-induced ovarian damage. We aimed to evaluate associations between previously identified genetic polymorphisms in CYP450 enzymes and AA-related ovarian function among adult CCSs. Methods: Anti-Müllerian hormone (AMH) levels served as a proxy for ovarian function in a discovery cohort of adult female CCSs, from the pan-European PanCareLIFE cohort (*n* = 743; age (years): median 25.8, interquartile range (IQR) 22.1–30.6). Using two additive genetic models in linear and logistic regression, nine genetic variants in three CYP450 enzymes were analyzed in relation to cyclophosphamide equivalent dose (CED) score and their impact on AMH levels. The main model evaluated the effect of the variant on AMH and the interaction model evaluated the modifying effect of the variant on the impact of CED score on log-transformed AMH levels. Results were validated, and meta-analysis performed, using the USA-based St. Jude Lifetime Cohort (*n* = 391; age (years): median 31.3, IQR 26.6–37.4). Results: CYP3A4*3 was significantly associated with AMH levels in the discovery and replication cohort. Meta-analysis revealed a significant main deleterious effect (Beta (95% CI): −0.706 (−1.11–−0.298), *p*-value = 7 × 10^−4^) of CYP3A4*3 (rs4986910) on log-transformed AMH levels. CYP2B6*2 (rs8192709) showed a significant protective interaction effect (Beta (95% CI): 0.527 (0.126–0.928), *p*-value = 0.01) on log-transformed AMH levels in CCSs receiving more than 8000 mg/m^2^ CED. Conclusions: Female CCSs CYP3A4*3 carriers had significantly lower AMH levels, and CYP2B6*2 may have a protective effect on AMH levels. Identification of risk-contributing variants may improve individualized counselling regarding the treatment-related risk of infertility and fertility preservation options.

## 1. Introduction

Over the past decades, major improvements in the treatment of childhood cancer have resulted in five-year survival rates that exceed 80% [1]. Factors credited for these improvements include enhanced treatment stratification, combined modality therapy, and advances in supportive care [2]. The increasing population of childhood cancer survivors (CCSs) has a life-long risk of treatment-related adverse health effects, one of which is gonadal function impairment [3,4,5,6,7]. The use of alkylating agents and radiotherapy exposing the ovaries are well-described risk factors for treatment-related gonadal damage [8,9,10,11]. However, inter-individual variability in gonadal injury among survivors exposed to similar gonadotoxic cancer therapy suggests a role for genetic susceptibility [9,12,13]. Knowledge of such genetic susceptibility has already been translated to clinical practice in the prediction and management of anthracycline-induced cardiotoxicity [14], but not as yet for other late effects [15]. 

Cyclophosphamide and ifosfamide are among the most commonly used alkylating agents in childhood cancers, effective against a wide range of cancers, including neuroblastoma, osteosarcoma, soft tissue sarcoma, leukemia, and lymphoma. Cyclophosphamide is a prodrug, in which the activation to 4-hydroxycyclophosphamide (4-OH-CPA) is catalyzed by the hepatic cytochrome P450 (CYP) isozymes including CYP2A6, 2B6, 3A4, 3A5, 2C9, 2C18, and 2C19 [16]. The highest 4-hydroxylase activity is displayed by CYP2B6. Thereafter, 4-OH-CPA forms the active phosphoramide mustard without enzymatic involvement [16]. As with cyclophosphamide, the metabolism of ifosfamide is required for the formation of the biologically active species. The metabolism of ifosfamide parallels that of cyclophosphamide, but with some differences in isozyme specificities and reaction kinetics [17].

Single nucleotide polymorphisms (SNPs) in CYP genes are associated with cyclophosphamide metabolism and toxic effects on ovarian function including impaired fertility and premature ovarian insufficiency (POI) in adult premenopausal women, but this has not been studied in exposed children with cancer [18,19]. Shu et al. evaluated the effect of SNPs in *CYP2B6* and *CYP2C19* on the pharmacokinetics of cyclophosphamide in Chinese patients treated for systemic lupus erythematosus. Their investigation identified star-alleles CYP2C19*2 and CYP2B6*1G that significantly influenced the 4-OH-CPA concentration; moreover, the combination of these two SNPs was significantly associated with short-term outcomes and side effects [18]. A study in breast cancer survivors receiving cyclophosphamide-based therapy by Su et al. evaluated time to chemotherapy-related ovarian failure and the effect of five SNPs in *CYP3A4*, *CYP2B6*, and *CYP3A5*. Patients with the CYP3A4*1B variant who were aged <45 years when receiving treatment showed a significantly longer time to ovarian failure than patients homozygote for CYP3A4*1A [19]. Ngamjanyaporn et al. observed that patients with the CYP2C19*1/*1 genotype had an increased risk of cyclophosphamide-related toxicity compared with carriers of the CYP2C19*2 allele [20]. Additionally, functional SNPs in drug-metabolizing enzymes (DME) have been shown to be associated with cancer-related outcomes [21,22,23,24]. Therefore, we hypothesized that genetic variation in DMEs that involves the metabolism of alkylating agents may be associated with the risk of long-term gonadal damage in CCSs [8,9,10,11,25].

Thorough evaluation of possible genetic determinants mandates large cohorts and independent replication cohorts. Here, we aimed to evaluate the associations between polymorphisms in candidate genes of CYP enzymes and treatment-related gonadal impairment that have been previously identified in adult patients receiving cyclophosphamide [18,19], in the largest European cohort of CCSs with available DNA [26], and to independently replicate findings within the St. Jude Lifetime Cohort Study (SJLIFE) cohort [27]. 

## 2. Methods 

### 2.1. Study Participants—Inclusion and Exclusion Criteria

Eligible participants were females diagnosed with cancer before the age of 25 years and treated with chemotherapy. They had survived at least five years after diagnosis, were ≥18 years of age at evaluation and provided a blood sample to quantify anti-Müllerian hormone (AMH) levels and for extraction of DNA. Exclusion criteria included: history of bilateral ovarian radiotherapy (defined as bilateral irradiation of the abdomen below the pelvic/iliac crest), central nervous system (CNS) irradiation, total body irradiation (TBI), or stem cell transplantation. Further details of the study protocol have been published previously [26].

### 2.2. Discovery Cohort

This international retrospective study is part of PanCareLIFE, a pan-European research project including 28 institutions from 13 countries addressing ototoxicity, fertility, and quality of life [8,28,29]. Demographic, disease, and treatment data were abstracted from medical records. Approval was obtained from all relevant local review boards in 13 countries and written informed consent was obtained from all participants. The study was conducted in accordance with the Declaration of Helsinki.

### 2.3. Replication Cohort

Female CCSs enrolled in the SJLIFE study served as the replication cohort. SJLIFE is a retrospectively-constructed cohort with prospective follow-up of patients diagnosed and treated at St. Jude Children’s Research Hospital (Memphis, TN, USA) between 1962–2012 and includes detailed treatment data, clinical assessment, patient-reported outcomes, whole-genome germline sequencing, and collection of biospecimens for research [30]. SJLIFE participants included in this analysis were female CCSs ≥10 years following cancer diagnosis and ≥18 years of age at clinical assessment, which included a comprehensive endocrinological evaluation. The inclusion and exclusion criteria applied to the discovery cohort were also applied to the SJLIFE replication cohort. The SJLIFE protocol is approved by the SJCRH Institutional Review Board. 

### 2.4. Outcome Definition

The outcome of this study was serum level of AMH reflecting ovarian function. AMH levels of both the discovery and replication cohort were determined in the same endocrine laboratory (VU University Medical Center in Amsterdam, the Netherlands) using an ultra-sensitive Elecsys AMH assay (Roche Diagnostics GmbH, Mannheim, Germany), as previously described [12]. Intra-assay coefficient of variation (CV) was 0.5–1.8%, the limit of detection (LoD) is 0.01 µg/L and limit of quantitation (LoQ) 0.03 µg/L [31]. 

### 2.5. Genotyping

Details about DNA processing are described in the Supplementary Material. For this study, SNPs were selected based on a literature search of published studies that identified cytochrome P450 (CYP) enzyme polymorphisms, associated with gonadotoxicity after treatment with alkylating agents such as cyclophosphamide [18,19,20]. Multiple SNPs in three CYPs were reported to have a significant effect on the enzymatic activity and therefore influence the gonadotoxic effect of alkylating agents. In the discovery cohort, we included all star-alleles of these three CYPs available in our genome-wide array or after imputation. Star-alleles are used to standardize genetic polymorphism annotation for CYP450 genes. The included polymorphisms were CYP2C19*2 (rs4244285), CYP2C19*17 (rs12248560), CYP3A4*1B (rs2740574), CYP3A4*3 (rs4986910), CYP3A4*22 (rs35599367), CYP2B6*2 (rs8192709), CYP2B6*6 (rs2279343), CYP2B6*9 (rs3745274), and CYP2B6*1G (rs4802101). Details of genotype data and the quality control protocol are provided in the Supplementary Material.

### 2.6. Alkylating Agents

For each survivor, the administered cumulative dose of alkylating agents was quantified using the Cyclophosphamide Equivalent Dose (CED)-score [32]. To evaluate the effects of no, low, medium, and high dose alkylating agent exposure, the CED score was divided into four categories (0; >0–4000 mg/m^2^; ≥4000–8000 mg/m^2^; ≥8000 mg/m^2^) [32], as previously described [12].

### 2.7. Statistical Analyses

Associations of the nine SNPs in the three CYP genes with chemotherapy-induced ovarian impairment were evaluated using AMH. We conducted a linear regression with log-transformed AMH, to adjust for the skewed residuals distribution of AMH, adjusted for age at time of serum sampling (Table S1, Figures S1–S3), CED score (none, >0–4000; ≥4000–8000; ≥8000 mg/m^2^) and 10 genetic principal components to account for ancestry. Additionally, we conducted a logistic regression analysis, adjusted for CED score (none, >0–4000; ≥4000–8000; ≥8000 mg/m^2^) and principal components. For the logistic regression analysis, cases and controls for gonadal impairment were defined as low versus high AMH levels per age category (≥18–25; ≥25–32; ≥32–40; ≥40 years), respectively. We defined cases and controls based on tertiles (the lower versus the highest tertile) and on a standard deviation (SD) based threshold (<−1.5 SD or <−2.0 SD versus above the threshold) (Supplementary Material). In the tertiles approach, one-third of the population will be excluded from the analysis, in the SD approach all survivors remain in the analysis. 

The modifying effect of SNPs on the impact of CED score on gonadal impairment was also investigated. The association between the selected SNPs and reduced ovarian function is based on two models. The main effect model (1) was adjusted for age at time of serum sampling, CED score, and genetic principal components. We fit an interaction model (2) that, aside from the terms included in the main effect model, additionally included an interaction term (SNP*CED category) for the genetic variant and CED score categories to evaluate the modifying effect of the variant on the impact of CED score on the log-transformed AMH levels.

Results of linear and logistic regression analyses are presented as regression coefficients (beta) with standard errors (SE) and odds ratios (OR) with a 95% confidence interval (95% CI), respectively. Sensitivity analyses performed to assess the robustness of our findings are provided in the Supplementary Materials.

SNPs that showed a statistically significant (*p*-values < 0.05) association with log-transformed AMH levels or reduced ovarian function in either model or an interaction effect with CED were selected for replication of both models. Statistical analyses were conducted using Statistical Package for Social Sciences (SPSS) version 24.0.0.1 and R version 3.5.1.

### 2.8. Replication and Meta-Analysis

We evaluated findings from the PanCareLIFE cohort in the SJLIFE replication cohort using identical models. In addition, we combined the data from the discovery and replication cohort and performed a meta-analysis using R version 3.5.1, package “rmeta” [33]. Details on the heterogeneity in the meta-analysis are described in the Supplementary Appendix. In the meta-analysis, a Bonferroni correction for multiple testing was applied to determine significance. *p*-values < 0.0167 (0.05/3) were considered to be statistically significant. The SNPs within one CYP are not independent, as the presence or absence of one or multiple SNPs in the CYP leads to changed activity/transcription and therefore a possible effect on the gonadotoxicity of chemotherapy. 

Finally, for all SNPs with a significant main effect, we calculated the cumulative betas for every genotype per CED category to allow interpretation of the findings for clinical applicability. The calculation reflects the expected difference in AMH for each genotype and every CED category compared to the AMH level of survivors with a wild-type genotype treated without alkylating agents. To this end, we back-transformed the betas of the logAMH.

## 3. Results

### 3.1. Discovery Cohort

In total, 743 CCSs from the PanCareLIFE cohort were included in the discovery cohort (Table 1). The median age at childhood cancer diagnosis was 8.3 years (interquartile range (IQR) (years) 3.3–14.0) and age at time of serum sampling was 25.8 years (IQR (years) 22.1–30.6). The median time since diagnosis was 18.3 years (IQR (years) 13.2–22.9). The most frequent diagnoses were leukemia (29.7%) and Hodgkin lymphoma (18.3%), followed by renal tumors (9.7%) and non-Hodgkin lymphoma (9.4%). The majority (64.5%) had not received any radiotherapy. Radiation of the thorax (14.8%) was the most common type of radiotherapy. Among cohort members, 35.8% received no alkylating agents, 24.6% received CED scores < 4000 mg/m^2^, 15.9% received ≥ 4000–8000 mg/m^2^, and 23.7% received ≥ 8000 mg/m^2^. All SNPs were in Hardy–Weinberg equilibrium (significance level < 1 × 10^−7^). Table S2 summarizes the alkylating agents received by participants and Table S3 displays pharmacokinetic information on these alkylating agents. Table 2 and Table S4 show the results of the linear regression in the discovery cohort including allele frequencies of the investigated SNPs. The analyses showed a significant negative main effect of presence of a polymorphism in CYP3A4*3 (rs4986910) on log-transformed AMH levels (beta −0.625, SE 0.252; *p*-value = 0.013). Eight patients were homozygous for this allele and, therefore, while the effect is statistically significant, it only affects few patients. Moreover, a significant interaction effect was seen with ≥ 4000–8000 mg/m^2^ and ≥ 8000 mg/m^2^ CED score, based on three patients (Table 2 and Table S4). Two SNPs showed a significant interaction effect with the group exposed to ≥8000 mg/m^2^ CED. CYP2C19*17 (rs12248560) showed a negative interaction effect with the presence of an alternative allele T (beta −0.240, SE 0.107; *p*-value = 0.025) and CYP2B6*2 (rs8192709) (beta 0.489, SE 0.227; *p*-value = 0.031) showed a positive interaction effect. The logistic regression based on tertiles did not show any significant effects (Table S5). However, when using standard deviations of log-transformed AMH to classify cases and controls for logistic regression, the same significant main effect of CYP3A4*3 is seen as was observed in the linear regression (Table S6). No interaction effect could be calculated for CYP3A4*3 due to a lack of survivors in the low AMH group, with the SNP and with a CED of 0 (Table S6). The effect of CYP3A4*3 is seen more clearly in a population receiving cyclophosphamide as part of the treatment regimen, which is extensively metabolized by CYP450 enzymes (Table S7a) and also when linear CED score is used in the analysis. Most sensitivity analyses performed to assess the choices of the model did not improve the model in the discovery cohort (Table S7b and Tables S8–S10). Three SNPs CYP2C19*17, CYP3A4*3, and CYP2B6*2 were selected for replication of the main and interaction linear regression model in the independent SJLIFE cohort. 

### 3.2. Replication Cohort

The replication cohort included 391 survivors who fulfilled the inclusion criteria, selected from 1644 female survivors in the SJLIFE cohort (Table 1). The median age at childhood cancer diagnosis was 6.9 years (IQR (years) 3.1–13.4) and age at study evaluation was 31.3 years (IQR (years) 26.6–37.4). The median time since diagnosis was 23.7 years (IQR (years) 18.3–29.3). The most frequent diagnosis was leukemia (30.9%). Hodgkin lymphoma (12.3%), neuroblastoma (9.2%), and central nervous system tumor (7.2%) were the next most common tumor groups. Among replication cohort members, 50.6% received no alkylating agents and 68.5% did not receive any radiotherapy (Table 1). In the replication cohort the main effect of the CYP3A4*3 was replicated (Beta −0.88, SE 0.37; *p*-value 0.02) (Table 3 and Table S11). 

### 3.3. Meta-Analysis

In the meta-analysis, CYP3A4*3 remained significantly associated with decreased log AMH levels in the main model (Beta −0.706, 95%CI −1.11–−0.298); *p*-value 7 × 10^−4^) (Table 3). In the interaction model (model 2), the groups became very small and the interaction effect of CYP3A4*3 was driven by a few individuals. The main model was thus more robust and used to create Table 4. In addition, the interaction of the polymorphism in CYP2B6*2 with CED exposure was significantly associated with log-transformed AMH levels (Table 3). While this interaction effect had not been statistically significant in the replication cohort, it did show the same trend as in the discovery cohort. Female CCSs who had received alkylating agents showed a decreasing trend in AMH levels. This effect increased in the presence of one alternative allele of rs4986910 in CYP3A4*3 compared to no alternative alleles. CCSs without the alternative allele of CYP3A4*3 receiving ≥8000 mg/m^2^ CED score have an estimated relative AMH level of 0.214 compared to someone receiving 0 mg/m^2^ CED score (Table 4). However, CCSs with the alternative allele of CYP3A4*3 receiving 0 mg/m^2^ CED score had an estimated relative AMH level of 0.197 and in those receiving ≥8000 mg/m^2^ CED score, this decreased to an estimated relative AMH level of 0.042. In the two cohorts, no homozygous carriers of the alternative allele were identified. 

## 4. Discussion

To our knowledge, this is the first study to assess the influence of pharmacogenetic factors on alkylating chemotherapy-induced impairment of ovarian function in female CCSs. Using AMH levels as a derivative biomarker of ovarian function, incorporating an identically phenotyped replication cohort as well as performing a meta-analysis, we identified an association between ovarian function among CCSs exposed to gonadotoxic therapy and a SNP in the *CYP3A4* gene leading to the CYP3A4*3 variant (rs4986910) [34]. An additional interaction effect of rs4986910 with alkylating agents on AMH levels was not observed in our cohorts. The alternative allele, which is present in 1.1% of Caucasians [34], results in a missense mutation with a Met445Thr substitution in exon 12 near the heme-binding region [35]. The CYP3A4*3 variant has not yet been associated with increased metabolism of alkylating agents, however, one study observed a significantly increased relative clearance of sildenafil in vitro [36]. This is in line with our finding of an increased gonadotoxic effect, indicated by the association of CYP3A4*3 with decreased AMH levels [34]. Nevertheless, the exact functionality of rs4986910 is still unknown. However, more evidence is available for other polymorphisms in *CYP3A4*. Similarly, the effect of the presence of the CYP3A4*1B allele is controversial. In our study, CYP3A4*1B had no significant effect on AMH. The presence of the alternative allele has been previously associated with increased ovarian toxicity in breast cancer survivors receiving cyclophosphamide as part of the treatment regimen [19] and in vitro with increased *CYP3A4* expression [37,38]. In contrast, carrying the CYP3A4*1B allele has also been associated with poorer survival in breast cancer patients receiving cyclophosphamide-containing therapy [22,39,40,41]. Of note, linkage of the CYP3A4*1B allele with the CYP3A5*1 expressor allele has previously been suggested based on increased total CYP3A activity (rather than increased CYP3A4 activity) [42,43,44,45,46]. Table S12 displays detailed information about the pharmacogenetics of the nine SNPs investigated. The cumulative betas for every genotype per CED category allow for better clinical interpretation and our data suggest that women of the same age receiving ≥4000–8000 mg/m^2^ CED are expected to have half (0.585-fold) the level of AMH compared to women receiving 0 mg/m^2^ CED (Table 4). However, if a woman carries the alternative (T) allele of rs4986910, a further five-fold decrease in AMH levels is expected, indicative of decreased ovarian reserve and a higher risk of POI (Table 4).

We also analyzed the effect of two other alleles on ovarian function. A protective effect of the presence of the CYP2B6*2 allele (rs8192709) has not previously been associated with gonadotoxicity. Yet, rs8192709 is in high linkage disequilibrium (LD) (EUR: D’ 0.9729) with the poor metabolizer rs4802101 (CYP2B6*1G) in both Caucasian and Asian populations (D’ = 0.894) [18]. This may explain the protective effect of the CYP2B6*2 allele in interaction with CED scores of ≥8000 mg/m2. The presence of rs4802101 (CYP2B6*1G) was not significantly associated with AMH levels in our cohort (*p*-value 0.068). The intronic variant CYP2C19*17 has an effect on promotor activity and is correlated with increased CYP2C19 activity [47,48,49,50]. However, the association of this SNP with AMH levels was not confirmed in our replication cohort, hence the effect in CCSs is unclear. 

Cyclophosphamide and ifosfamide are both extensively metabolized by *CYP2B6* and *CYP2C19* and to a lesser extent by CYP3A4 to their active metabolites. CYP3A4 is also involved in the formation of dechloromethyl-CPA (Figure S4) [25]. Thus, in the presence of a defective CYP450 enzyme, the other enzymes can compensate for the loss of function. The redundancy of CYP enzymes in the metabolic pathway of alkylating agents such as cyclophosphamide or ifosfamide may reduce the functional effect of polymorphism in a single CYP450 enzyme. For star-alleles with only a small effect on pharmacokinetics, the presence of multiple altered CYPs may be required to lead to a clinically relevant change in the effect of alkylating agents on gonadotoxicity. 

In contrast to the linear regression, the logistic regression analysis limited to participants with especially high or low AMH values (tertiles) in the discovery cohort showed no significant effects on AMH levels. Yet, when using a standard deviation-based threshold of AMH to classify cases and controls for logistic regression, a significant main effect of CYP3A4*3 was seen, yielding similar results to the linear regression (Table S6). This may result from our comparison of the lowest AMH tertile with the highest AMH, excluding the middle tertile in the first logistic regression. Whereas in the analysis with standard deviations, no participants were excluded and thus the power was not reduced. Thus, the first analysis was restricted by a relatively small sample size, yet the contrast between the groups was higher. 

One genome-wide association study (GWAS) in CCSs using data from the SJLIFE and the Childhood Cancer Survivor Study identified a haplotype formed by 4 of 13 SNPs, located upstream of the neuropeptide receptor 2 gene (*NPY2R*) associated with premature menopause. The prevalence of premature menopause was increased in homozygous carriers of this haplotype after exposure to ovarian radiotherapy [51]. We previously reported and replicated a modifying effect of a polymorphism in the *BRSK1* gene on the association between alkylating agents and AMH levels [9,12]. AMH, produced by the granulosa cells of small growing follicles in the ovaries, has been found to reflect the gradual decline in reproductive capacity with increasing age [52] and can serve as an early, sensitive marker of reduced ovarian function in young cancer survivors [53,54] and as an early predictor of time to menopause [55,56,57]. However, the relationship between AMH and time to menopause on an individual level remains complex [58,59]. So far, large-scale GWAS have identified several SNPs, such as rs11668344 (*BRSK1*), rs365132 (*UIMC1*) and rs16991615 (*MCM8*), relevant for age at natural menopause or premature ovarian insufficiency (POI) in the general population [12,60,61,62,63,64,65]. Results of a European GWAS study in CCSs exploring genetic susceptibility of cancer treatment-related gonadal damage in girls are currently pending [26,28].

AMH is highly correlated with antral follicle count (AFC) in a healthy population [66,67,68,69,70]. AMH production is gonadotropin independent. Thus, AMH levels are less affected by the normal menstrual cycle than FSH, LH, and oestradiol, which are menstrual cycle-dependent, adding an extra challenge to the clinical testing of gonadal impairment [71]. Additionally, AMH is currently used in predicting the oocyte yield after ovarian stimulation in assisted reproductive technology [59]. 

This study presents results from the largest cohort of female CCSs with available AMH levels and DNA samples and replicates findings in a large representative childhood cancer cohort. In addition, this is the first study to evaluate the effect of SNPs in CYP450 enzymes on gonadotoxicity of exposure to alkylating agents in CCSs. Moreover, all AMH samples including those of the replication cohort were processed at the same laboratory ensuring consistent and comparable results. As we have categorized survivors on CED score instead of tumor type in our analyses, we can generalize the findings of this study towards patients treated in the future, even though some survivors received therapeutic strategies that are no longer in use. A limitation of our study is that the discovery cohort and replication cohort were not completely comparable regarding the distribution of CED categories. However, this variability in the cohort also makes our results more generalizable. Moreover, the main effect of CYP3A4*3 is based on a small number of participants, eight participants in the discovery and 12 in the replication cohort with the alternative allele. Due to the small numbers, no conclusions could be drawn from the interaction model of CYP3A4*3 and thus the main model was used to create Table 4 and draw final conclusions. Furthermore, the limited number of survivors with alternative alleles reduced the power of our statistical analyses. Another limitation of the candidate gene approach is that only known variants are included in the analysis. A GWAS does not have this limitation and would be a valuable next step to uncover novel variants associated with inter-individual differences in gonadal damage after childhood cancer treatment. Finally, as a result of the “winner’s curse”, our findings may overestimate the genetic effect and follow-up studies may show a smaller effect when replicating our findings [72,73].

Our findings may enhance clinical practice by identifying patients at high risk of ovarian impairment who more than others may benefit from referral and counseling about fertility preservation options. Recently, the International Guideline Harmonisation Group (IGHG) published recommendations advising that all patients should be informed about their potential risk of gonadal damage [74,75,76]. This is in line with the current views and wishes of both healthcare providers and patients and their families [77,78]. For girls at high risk of infertility, preservation measures can be considered. In most cases, this involves the surgical removal of an ovary, which carries small risks of infection and bleeding. Cryopreservation of oocytes is only possible in some older adolescent patients, who can postpone cancer treatment. Although the age at menopause is found to occur only a few years earlier after unilateral ovariectomy [79,80,81], the procedure reduces the in situ ovarian reserve [81]. Therefore, it is of utmost importance to be able to determine who is at risk [82], which may include consideration of genetic factors in risk estimation in the near future. Pharmacogenetics is increasingly used in adjusting therapy to the personalized optimal dose for the patient and minimizing side effects [83]. Future prospective research is important to establish the clinical relevance of pharmacogenetic polymorphisms in estimating the gonadotoxic effect of cancer treatment. In addition, differences in pharmacokinetics between children and adults may play a role and require further study [84,85,86,87]. Evidence-based knowledge of genetic predisposition to alkylating agent toxicity may optimize the delivery of personalized therapy and fertility counseling in the future [2]. 

## 5. Conclusions

In conclusion, adult female CCSs carrying the CYP3A4*3 allele appear to have a five-fold lower ovarian function after treatment for childhood cancer, and CYP2B6*2 may have a protective effect on AMH levels in patients receiving CED scores of 8000mg/m^2^ or more. Identifying pharmacogenetic risk factors for alkylating agent-related gonadotoxicity such as the presence of a CYP3A4*3 allele may improve risk prediction models for reduced ovarian function and consequent infertility in female CCSs. This may lead to a better identification of patients at high risk to whom fertility preservation options may be offered. Upfront fertility preservation programs, including ovarian tissue cryopreservation, would benefit from optimized prediction models to identify paediatric cancer patients at the highest risk for gonadotoxicity for whom the balance of benefits of fertility preservation—including ethical considerations—likely outweighs harms [88].

## Figures and Tables

**Table 1:** Characteristics of participating CCSs of the discovery PanCareLIFE cohort and CCSs of the replication St. Jude LIFE cohort (SJLIFE).

Characteristics	Discovery PanCareLIFE Cohort (*n* = 743)	Replication SJLIFE (*n* = 391)
Age at time of study (years) Median (IQR)	25.8 (22.1–30.6)	31.3 (26.6–37.4)
Age at diagnosis (years) Median (IQR)	8.3 (3.3–14.0)	6.9 (3.1–13.4)
Time since diagnosis (years) Median (IQR)	18.3 (13.2–22.9)	23.7 (18.3–29.3)
Diagnosis		
− Leukemia	221 (29.7%)	121 (30.9%)
− Hodgkin lymphoma	136 (18.3%)	48 (12.3%)
− Non-Hodgkin lymphoma	70 (9.4%)	22 (5.6%)
− Brain tumour	17 (2.3%)	28 (7.2%)
− Neuroblastoma	46 (6.2%)	36 (9.2%)
− Renal tumor	72 (9.7%)	27 (6.9%)
− Carcinoma (hepatic, thyroid, colon, liver, other)	7 (0.9%)	9 (2.3%)
− Osteosarcoma	33 (4.4%)	22 (5.6%)
− Ewing sarcoma	31 (4.2%)	12 (3.1%)
− Soft tissue sarcoma	49 (6.6%)	18 (4.6%)
− Germ cell tumour	34 (4.6%)	13 (3.3%)
− Skin cancer (incl. melanoma)	3 (0.4%)	1 (0.3%)
− Retinoblastoma	5 (0.7%)	20 (5.1%)
− Other	12 (1.6%)	3 (0.8%)
− Non malignant	0	1 (0.3%)
Radiotherapy		
− No	479 (64.5%)	268 (68.5%)
− Yes *	264 (35.5%)	123 (31.5%)
− Thorax	110 (14.8%)	71 (18.2%)
− Spine	5 (0.7%)	6 (1.5%)
− Abdomen, not pelvic	15 (2.0%)	30 (7.7%)
− Unilateral pelvis	9 (1.2%)	3 (0.8%)
− Other	78 (10.5%)	51 (13.0%)
CED score		
− 0	266 (35.8%)	198 (50.6%)
− >0–4000	183 (24.6%)	21 (5.4%)
− ≥4000–8000	118 (15.9%)	78 (19.9%)
− ≥8000	176 (23.7%)	94 (24.0%)
Unilateral surgery of ovary		
− No	740 (99.6%)	391 (100.0%)
− Yes	3 (0.4%)	0
Anti-Müllerian hormone level		
Median (IQR)	2.33 (1.02–4.03)	1.84 (0.68–3.28)
Age category 18–25 (IQR)	2.70 (1.41–4.39)	2.79 (1.68–4.14)
Age category ≥25–32 (IQR)	2.62 (1.37–4.24)	2.55 (1.44–3.90)
Age category ≥32–40 (IQR)	1.22 (0.41–2.58)	1.69 (0.70–2.55)
Age category ≥40 (IQR)	0.27 (0.13–0.52)	0.09 (0.01–0.47)

* Not mutually exclusive. Values represent the number (%) of women unless indicated otherwise. IQR = interquartile range (25th–75th percentile); CED score = Cyclophosphamide Equivalent Dose Score; CCSs = childhood cancer survivors; AMH, anti-Müllerian hormone in μg/L.

**Table 2:** Results of the linear regression based on log-transformed AMH and interaction in the Discovery cohort PanCareLIFE.

Gene	Variant	Star-allele	Model	Variant, Interaction	*n* (0/1/2) ‡	Beta (SE)	*p*-Value
CYP2C19	rs4244285	*2	1	rs4244285	536/189/18	−0.019 (0.047)	0.692
			2	rs4244285		0.025 (0.081)	0.756
				SNP*CED: 0	200/60/6	0 (ref) †	0.857 ^
				>0–4000	129/50/4	−0.107 (0.124)	0.386
				≥4000–8000	89/25/4	−0.051 (0.141)	0.718
				≥8000	118/54/4	−0.034 (0.124)	0.784
CYP2C19	rs12248560	*17	1	rs12248560	432/274/37	−0.017 (0.041)	0.674
			2	rs12248560		0.062 (0.068)	0.366
				SNP*CED: 0	161/92/13	0 (ref) †	0.150 ^
				>0–4000	99/77/7	−0.056 (0.108)	0.605
				≥4000–8000	67/44/7	−0.047 (0.119)	0.691
				≥8000	105/61/10	−0.240 (0.107)	0.025
CYP3A4	rs2740574	*1B	1	rs2740574	690/53/0	−0.004 (0.093)	0.963
			2	rs2740574		−0.049 (0.152)	0.748
				SNP*CED: 0	246/20/0	0 (ref) †	0.243 ^
				>0–4000	165/18/0	0.166 (0.222)	0.455
				≥4000–8000	114/4/0	0.520 (0.364)	0.154
				≥8000	165/11/0	−0.202 (0.251)	0.420
CYP3A4	rs4986910	*3	1	rs4986910	735/8/0	−0.625 (0.252)	0.013
			2	rs4986910		0.185 (0.515)	0.719
				SNP*CED: 0	264/2/0	0 (ref) †	0.015 ^
				>0–4000	180/3/0	−0.317 (0.655)	0.629
				≥4000–8000	116/2/0	−1.558 (0.740)	0.035
				≥8000	175/1/0	−2.195 (0.821)	0.008
CYP3A4	rs35599367	*22	1	rs35599367	678/62/3	−0.001 (0.080)	0.988
			2	rs35599367		0.006 (0.131)	0.966
				SNP*CED: 0	241/24/1	0 (ref) †	0.465 ^
				>0–4000	169/14/0	−0.244 (0.223)	0.274
				≥4000–8000	106/11/1	0.038 (0.219)	0.861
				≥8000	162/13/1	0.137 (0.210)	0.515
CYP2B6	rs8192709	*2	1	rs8192709	678/63/2	0.047 (0.081)	0.560
			2	rs8192709		−0.020 (0.116)	0.860
				SNP*CED: 0	237/27/2	0 (ref) †	0.093 ^
				>0–4000	167/16/0	0.038 (0.206)	0.855
				≥4000–8000	110/8/0	−0.209 (0.263)	0.428
				≥8000	164/12/0	0.489 (0.227)	0.031
CYP2B6	rs2279343	*6	1	rs2279343	410/279/54	−0.038 (0.039)	0.327
			2	rs2279343		−0.077 (0.064)	0.225
				SNP*CED: 0	147/98/21	0 (ref) †	0.696 ^
				>0–4000	106/67/10	0.118 (0.104)	0.256
				≥4000–8000	58/50/10	0.057 (0.115)	0.621
				≥8000	99/64/13	0.014 (0.102)	0.891
CYP2B6	rs3745274	*9	1	rs3745274	426/269/48	−0.045 (0.039)	0.250
			2	rs3745274		−0.083 (0.064)	0.197
				SNP*CED: 0	154/94/18	0 (ref) †	0.562 ^
				>0–4000	111/64/8	0.138 (0.105)	0.188
				≥4000–8000	58/51/9	0.047 (0.114)	0.679
				≥8000	103/60/13	0.001 (0.101)	0.991
CYP2B6	rs4802101	*1G	1	rs4802101	118/336/289	−0.006 (0.034)	0.857
			2	rs4802101		−0.083 (0.056)	0.142
				SNP*CED: 0	43/118/105	0 (ref) †	0.383 ^
				>0–4000	32/88/63	0.125 (0.089)	0.160
				≥4000–8000	11/63/44	0.085 (0.112)	0.445
				≥ 8000	32/67/77	0.133 (0.087)	0.127

‡ n = alternative allele frequency is reported as 0/1/2 (recalculated based on allelic dosage), other analyses are performed with allelic dosage. † reference is corresponding rs*CED 0 (ref). ^ the reported *p*-value is the overall *p*-value for the interaction analysis in model 2. The multivariable model 1 is adjusted for 10 principal components (PC), CED score, and age. Model 2 additionally includes an interaction term (SNP*CED category) for the genetic variant and CED score categories to evaluate the modifying effect of the variant on the impact of CED score on low AMH levels. Model 1: crude effect of variant, crude effect of CED categories, corrected for 10PC and age. Model 2: linear regression based on log-transformed AMH and interaction. Multivariable model adjusted for principal components, CED score, and age. AMH = anti-Müllerian hormone, CED = cyclophosphamide equivalent dose.

**Table 3:** Results of the linear regression based on log-transformed AMH and interaction of the Discovery cohort PanCareLIFE, Replication cohort SJLIFE, and meta-analysis.

Gene	Variant	Star-allele	Model	Variant, Interaction	Discovery Cohort PanCareLIFE		Replication Cohort SJLIFE		Discovery + Replication Meta-Analysis		
					Beta (SE)	*p*-Value	Beta (SE)	*p*-Value	Beta (95% CI)	*p*-Value	Heterogeneity and *p*-Value
CYP3A4	rs4986910	*3	1	rs4986910	−0.625 (0.252)	0.013	−0.88 (0.37)	0.02	−0.706 (−1.11–−0.298)	0.0007	0; 0.569
				CED: 0	0 (ref) †	6.51 × 10^−29^ ^	0 (ref) †	0.13 ^	0 (ref) †	0	
				>0–4000	−0.027 (0.063)	0.672	0.16 (0.29)	0.59	−0.019 (−0.139–0.102)	0.763	0; 0.529
				≥4000–8000	−0.234 (0.072)	0.001	−0.23 (0.17)	0.17	−0.233 (−0.363–−0.103)	0.0004	0; 0.983
				≥8000	−0.728 (0.065)	2.69 × 10^−27^	−0.31 (0.16)	0.05	−0.669 (−0.787–−0.551)	1.18 × 10^−28^	0.83; 0.016
			2	rs4986910	0.185 (0.515)	0.719	−0.81 (0.52)	0.12	−0.308 (−1.02–0.409)	0.400	0.45; 0.174
				CED: 0	0 (ref) †	9.83 × 10^−28^ ^	0 (ref) †	0.15 ^	0 (ref) †	0	
				>0–4000	−0.027 (0.063)	0.663	0.15 (0.30)	0.62	−0.020 (−0.14–0.101)	0.751	0; 0.564
				≥4000–8000	−0.215 (0.072)	0.003	−0.21 (0.17)	0.22	−0.214 (−0.344–−0.084)	0.001	0; 0.978
				≥8000	−0.712 (0.064)	2.71 × 10^−26^	−0.32 (0.16)	0.05	−0.658 (−0.774–−0.541)	1.71 × 10^−28^	0.81; 0.023
				SNP*CED: 0	0 (ref) †	0.015 ^	0 (ref) †	0.82 ^	0 (ref) †	0.066 ^	
				>0–4000	−0.317 (0.655)	0.629	0.20 (1.38)	0.88	−0.222 (−1.38–0.938)	0.708	0; 0.735
				≥4000–8000	−1.558 (0.740)	0.035	−0.46 (0.84)	0.58	−1.08 (−2.17–0.0101)	0.052	0; 0.327
				≥8000	−2.195 (0.821)	0.008	0.83 (1.36)	0.54	−1.39 (−2.76–−0.009)	0.048	0.72; 0.057
CYP2B6	rs8192709	*2	1	rs8192709	0.047 (0.081)	0.560	0.06 (0.18)	0.74	0.049 (−0.096–0.194)	0.505	0; 0.947
				CED: 0	0 (ref) †	1.69 × 10^−28^ ^	0 (ref) †	0.15 ^	0 (ref) †	0	
				>0–4000	−0.030 (0.063)	0.637	0.15 (0.29)	0.62	−0.022 (−0.143–0.099)	0.722	0; 0.544
				≥4000–8000	−0.238 (0.072)	0.001	−0.25 (0.17)	0.14	−0.240 (−0.37–−0.11)	0.0003	0; 0.948
				≥8000	−0.727 (0.065)	5.59 × 10^−27^	−0.29 (0.16)	0.07	−0.665 (0.783–−0.547)	2.33 × 10^−28^	0.84; 0.011
			2	rs8192709	−0.020 (0.116)	0.860	−0.11 (0.29)	0.72	−0.032 (−0.244–0.179)	0.763	0; 0.773
				CED: 0	0 (ref) †	3.95 × 10^−29^ ^	0 (ref) †	0.09 ^	0 (ref) †	0	
				>0–4000	−0.037 (0.066)	0.579	0.14 (0.31)	0.64	−0.029 (−0.156–0.097)	0.650	0; 0.577
				≥4000–8000	−0.229 (0.075)	0.002	−0.24 (0.18)	0.18	−0.231 (−0.366–−0.095)	0.0009	0; 0.955
				≥8000	−0.765 (0.067)	1.50 × 10^−27^	−0.39 (0.17)	0.02	−0.715 (−0.837–−0.592)	2.00 × 10^−30^	0.76; 0.04
				SNP*CED: 0	0 (ref) †	0.093 ^	0 (ref) †	0.44 ^	0 (ref) †	0.172 ^	
				>0–4000	0.038 (0.206)	0.855	−0.09 (0.98)	0.92	0.0326 (−0.363–0.428)	0.872	0; 0.898
				≥4000–8000	−0.209 (0.263)	0.428	0.01 (0.40)	0.98	−0.143 (−0.574–0.288)	0.516	0; 0.647
				≥8000	0.489 (0.227)	0.031	0.69 (0.47)	0.14	0.527 (0.126–0.928)	0.010	0; 0.700
CYP2C19	rs12248560	*17	1	rs12248560	−0.017 (0.041)	0.674	−0.01 (0.11)	0.91	−0.016 (−0.091–0.059)	0.674	0; 0.952
				CED: 0	0 (ref) †	1.15 × 10^−28^ ^	0 (ref) †	0.15 ^	0 (ref) †	0	
				>0–4000	−0.030 (0.063)	0.631	0.13 (0.29)	0.65	−0.023 (−0.143–0.098)	0.711	0; 0.59
				≥4000–8000	−0.240 (0.072)	0.0009	−0.25 (0.17)	0.14	−0.242 (−0.371–−0.112)	0.0003	0; 0.957
				≥8000	−0.729 (0.065)	3.63 × 10^−27^	−0.30 (0.16)	0.06	−0.668 (−0.786–−0.55)	1.31 × 10^−28^	0.84; 0.013
			2	rs12248560	0.062 (0.068)	0.366	−0.15 (0.16)	0.38	0.030 (−0.093–0.152)	0.637	0.33; 0.223
				CED: 0	0 (ref) †	3.06 × 10^−14^ ^	0 (ref) †	0.15 ^	0 (ref) †	1.56 × 10^−13^ ^	
				>0–4000	0.007 (0.082)	0.934	−0.07 (0.34)	0.84	0.003 (−0.153–0.159)	0.972	0; 0.826
				≥4000–8000	−0.222 (0.092)	0.016	−0.28 (0.21)	0.17	−0.231 (−0.397–−0.066)	0.006	0; 0.80
				≥8000	−0.620 (0.081)	5.88 × 10^−14^	−0.44 (0.20)	0.03	−0.595 (−0.742–−0.447)	2.37 × 10^−15^	0; 0.404
				SNP*CED: 0	0 (ref) †	0.150 ^	0 (ref) †	0.53 ^	0 (ref) †	0.281 ^	
				>0–4000	−0.056 (0.108)	0.605	0.58 (0.52)	0.26	−0.030 (−0.237–0.178)	0.779	0.30; 0.231
				≥4000–8000	−0.047 (0.119)	0.691	0.08 (0.29)	0.78	−0.029 (−0.244–0.187)	0.794	0; 0.685
				≥8000	−0.240 (0.107)	0.025	0.30 (0.26)	0.26	−0.162 (−0.356–0.032)	0.102	0.729; 0.055

† reference is corresponding rs*CED 0 (ref). ^ the reported *p*-value is the overall *p*-value for the analysis. The multivariable model 1 is adjusted for 10 principal components (PC), Cyclophosphamide Equivalent Dose (CED) score, and age. Model 2 additionally includes an interaction term (SNP*CED category) for the genetic variant and CED score categories to evaluate the modifying effect of the variant on the impact of CED score on low AMH levels. Model 1 both cohorts: crude effect of variant, crude effect of CED categories, corrected for 10PC and age. Model 1 meta-analysis: LOGAMH = rsID + 10 Principal Components + Age + CEDcategories. Model 2 both cohorts: linear regression based on log-transformed AMH and interaction. Multivariable model adjusted for principal components, CED score, and age. Model 2 meta-analysis: LOGAMHint = rsID + 10 Principal Components + Age + CEDcategories + rsID *CEDcategories. Where LOGAMH is the log-transformed level of AMH, rsID is the genotype, Age is the Age at AMH sampling, CEDcategories are the Cyclophosphamide Equivalent Dose (CED) categories (None, >0–4000; ≥4000–8000; ≥8000 mg/m^2^). AMH = anti-Müllerian hormone, SE = standard error; CI = confidence interval.

**Table 4:** Estimated relative AMH levels per genotype of CYP3A4*3 (rs4986910) and CED score based on prevalence in two cohorts.

CED in mg/m^2^	Genotype TT		Genotype TC (CYP3A4*3)	
	*n* (1114)	Exp Beta (CI) AMH	*n* (20)	Exp Beta (CI) AMH
0	456	1 (ref)	8	0.197 (0.078–0.504)
>0–4000	200	0.958 (0.726–1.265)	4	0.189 (0.056–0.637)
≥4000–8000	190	0.585 (0.434–0.789)	6	0.115 (0.034–0.397)
≥8000	268	0.214 (0.163–0.281)	2	0.042 (0.013–0.142)

The results from model 1 of the meta-analysis in Table 3 were used to create this table. Genotype TT is the genotype of CYP3A4 without *3 and TC contains 1 allele of CYP3A4*3 (rs4986910). AMH = anti-Müllerian hormone, *n* = represents the number of cases within each genotype group. CED = Cyclophosphamide Equivalent Dose. CI = conservative confidence intervals. Exp Beta (CI) is calculated based on the prevalence of a reduced ovarian function for every genotype and every CED category compared to the prevalence of a reduced ovarian function for survivors with a TT genotype treated without alkylating agents. The exp beta calculation: 10^(beta of alt allele + beta of CED). The CI calculation: 10^(CI of alt allele + CI of CED).

## Data Availability

The data of this study are available upon request. The use of health-related data for research is constrained by data protection legislation, the common law duty of confidentiality, and in many cases, requirements set by research ethics committees. Every effort is made to perform research on data that are as “de-identified’ as possible. However, the present study required a very detailed data set comprising many variables about each person. While it is unlikely that any of the variables in isolation could be used to identify a given individual, it is possible that a combination of variables could render a person’s or persons’ record(s) potentially identifiable. Data from the PanCareLIFE cohort are available to members of the consortium within the first 5 years after ending data collection. Data from the St Jude Lifetime Cohort is available upon request via St Jude.

## Supplementary Materials

The following are available online at https://www.mdpi.com/article/10.3390/cancers13184598/s1, Supplementary Text, Table S1: Standard deviation (SD) scores per age category for log-transformed Anti-Müllerian Hormone (AMH), Table S2: Mono-therapies and Combination therapies in PanCareLIFE Discovery cohort, Table S3: Pharmacokinetics of alkylating agents, Table S4: Results linear regression based on log-transformed AMH and interaction for Discovery cohort PanCareLIFE, Table S5: Results logistic regression based on lowest tertile of AMH vs. highest tertile of AMH (N = 243 vs. N = 240) and interaction in Discovery cohort PanCareLIFE, Table S6a+b: Sensitivity analysis: Logistic regression based on low AMH (<−1.5SD or <−2.0SD) versus high AMH (≥−1.5SD or ≥−2.0SD) and interaction in Discovery cohort PanCareLIFE, Table S7a+b: Sensitivity analysis: linear regression only on patients receiving cyclophosphamide, Table S8: Sensitivity analysis correction for age at diagnosis, Table S9: Sensitivity analysis: correction for cyclophosphamide, Table S10: Sensitivity analysis: logistic regression tertiles per year, Table S11: Results of the replication analysis in the SJLIFE cohort, Table S12: Pharmacogenetics, Figure S1: Scatterplot AMH levels per age, Figure S2: Scatterplot SDscores of Log-transformed AMH levels per age, Figure S3: Distribution of SDscores of Log-transformed AMH and AMH, Figure S4: Cyclophosphamide major biotransformation pathways.
