# Supplementary material for: Effect of Genetic Variation in CYP450 on Gonadal Impairment in a European Cohort of Female Childhood Cancer Survivors, Based on a Candidate Gene Approach: Results from the PanCareLIFE Study
Source: Cancers (Basel). 2021 Sep 13;13(18):4598. doi: 10.3390/cancers13184598 (PMC8470074; doi:10.3390/cancers13184598)
Supplement: Supplementary file 1 [file cancers-13-04598-s001.zip › cancers-1332026-supplementary.pdf]

Supplementary Materials:

Supplementary Text.

Supplementary Tables:

Table S1. Standard deviation (SD) scores per age category for log-transformed Anti-Müllerian Hormone (AMH)
Table S2. Mono-therapies and Combination therapies in PanCareLIFE Discovery cohort
Table S3. Pharmacokinetics of alkylating agents
Table S4. Results linear regression based on log-transformed AMH and interaction for Discovery cohort PanCareLIFE
Table S5. Results logistic regression based on lowest tertile of AMH vs highest tertile of AMH (*N*=243 vs *N*= 240) and interaction in Discovery cohort PanCareLIFE
Table S6a+b. Sensitivity analysis: Logistic regression based on low AMH (< -1.5SD or <-2.0SD) versus high AMH (≥ -1.5SD or ≥-2.0SD) and interaction in Discovery cohort PanCareLIFE
Table S7a+b. Sensitivity analysis: linear regression only on patients receiving cyclophosphamide
Table S8. Sensitivity analysis correction for age at diagnosis
Table S9. Sensitivity analysis: correction for cyclophosphamide
Table S10. Sensitivity analysis: logistic regression tertiles per year
Table S11. Results of the replication analysis in the SJLIFE cohort
Table S12. Pharmacogenetics

Supplementary Figures:

Figure S1. Scatterplot AMH levels per age
Figure S2. Scatterplot SDscores of Log-transformed AMH levels per age
Figure S3. Distribution of SDscores of Log-transformed AMH and AMH
Figure S4. Cyclophosphamide major biotransformation pathways

Supplementary references

Supplementary text

Quality protocol

For the PanCareLIFE cohort, genomic DNA was extracted from whole blood samples and genotyping was performed using the Global Screening Array by Illumina, as previously described(1). A quality control (QC) protocol containing multiple filters was applied to clean the genetic data(2). Both a SNP and individual call rate filter of 97.5% were applied to remove poorly genotyped SNPs and individuals from the data. A Hardy-Weinberg Equilibrium test (significance level $<1x10^{-7}$) was employed to remove variants containing potential genotyping errors. Furthermore, samples with excess heterozygosity, gender mismatches and related samples were removed from the data. The majority of samples were from European Ancestry. Imputation was performed using the Michigan Imputation Server using default settings(3) with the Haplotype Reference Consortium (HRC r1.1) as reference panel(4). The same approach has previously been used in large-scale population studies such as the Rotterdam Study(5) and Generation R(6). Genotyping quality was double checked with the QuantStudio 7 Taqman for CYP3A4*3 (rs4986910).
For the St. Jude Lifetime Cohort study, genotyping was performed using Affymetrix HumanSNP6.0 array (Affymetrix Incorporated, Santa Clara, CA). Quality control (QC) of this genotype data was performed using PLINK, version 1.90 and was previously reported(7).

Statistical analyses

The candidate SNPs were tested for deviation from the Hardy-Weinberg equilibrium. By using an additive mode of inheritance, SNP genotypes were estimated based on the imputed dosage information and were coded according to the number of minor alleles as a value between 0 and 2.

Statistical analysis

For the logistic regression analysis, cases and controls for gonadal impairment were defined as follows: All women aged 18 until 40 years at time of serum sampling were divided in four age categories (≥18-25; ≥25-32; ≥32-40; ≥40 years) to account for age-dependency of AMH. The categories are based on patient numbers, driven by clinical relevance and to ensure sufficient power among the groups. Within these age categories, AMH values were divided into tertiles, with the exception of the oldest age category in which the AMH level variation was too small to adequately define tertiles. For the current analysis cases were defined as CCS with an AMH level in the lowest tertile for their age category, thus considered to have a reduced ovarian function. Controls were defined as CCS with an AMH-value in the highest tertile for their age category and thus were assumed not to have a reduced ovarian function. Women over 40 years of age were only considered a 'case' if they reported premature menopause (absence of menses for > 12 months before the age of 40) at time of study. No 'control' subjects were defined in this age group due to the inability to identify reduced normal ovarian function at that age.

Heterogeneity in the meta-analysis

Heterogeneity between the cohorts was assessed using the estimated heterogeneity variance with corresponding *P*-values. Low estimates of heterogeneity variance indicated sufficient similarity between cohorts indicating that pooling was not unreasonable. Pooled estimates based on the fixed effects model were presented. However, it should be considered that only two cohorts were compared.

Supplementary Tables:

Table S1. SD scores per age category for logAMH in the Discovery cohort

Age category (years)	SD scores	Frequency	%
≥18-25	<-2	18	5.5
	-2 - -1	6	1.8
	-1 - 0	76	23.2
	0 - 1	214	65.4
	1 - 2	13	4.0
	Total	327	100.0
≥25-32	<-2	11	4.3
	-2 - -1	8	3.1
	-1 - 0	70	27.1
	0 - 1	159	61.6
	1 - 2	10	3.9
	Total	258	100.0
≥32-41	<-2	14	10.0
	-2 - -1	8	5.7
	-1 - 0	25	17.9
	0 - 1	80	57.1
	1 - 2	13	9.3
	Total	140	100.0
≥41-60	<-2	1	5.9
	-2 - -1	2	11.8
	-1 - 0	5	29.4
	0 - 1	7	41.2
	1 - 2	1	5.9
	> 2	1	5.9
	Total	17	100.0

SD = standard deviation; logAMH = logtransformed Anti-Müllerian Hormone

Table S2. Mono-therapies and Combination therapies in both cohorts

Mono-/combination therapy	Discovery PanCareLIFE cohort (*N*=743)	Replication SJLIFE cohort (*N*=391)
**Monotherapy:**		
Cyclophosphamide	250	134
Ifosfamide	70	14
Chlorambucil	1	0
Procarbazine	2	0
Mechlorethamine	5	1
Lomustine	0	1
**Combination therapy:**		
Ifosfamide + Cyclophosphamide	48	9
Procarbazine + Cyclophosphamide	34	14
Mechlorethamine + Procarbazine	21	0
Chlorambucil + Procarbazine	10	0
Melphalan + Cyclophosphamide	8	0
Melphalan + Cyclophosphamide + Ifosfamide + Busulfan	8	0
Melphalan + Ifosfamide	4	0
Busulfan + Cyclophosphamide	0	2
Cyclophosphamide + Dacarbazine	0	2
Mechlorethamine + Cyclophosphamide	0	1
Melphalan + Ifosfamide + Busulfan	2	0
Procarbazine + Cyclophosphamide + Ifosfamide	2	0
Thiotepa + Cyclophosphamide	2	1
Thiotepa + Cyclophosphamide + Ifosfamide	2	0
Thiotepa + Cyclophosphamide + Melphalan + Busulfan	2	0
Carmustine + Cyclophosphamide + Melphalan + Ifosfamide	2	0
Carmustine + Cyclophosphamide	1	2
Mechlorethamine + Chlorambucil + Procarbazine	1	0
Melphalan + Cyclophosphamide + Busulfan	1	0
Thiotepa + Cyclophosphamide + Ifosfamide + Melphalan + Busulfan	1	0
Thiotepa + Cyclophosphamide + Busulfan	1	0
Cyclophosphamide + Procarbazine + Dacarbazine	0	8
Cyclophosphamide + Procarbazine + Mechlorethamine	0	2
Cyclophosphamide + Procarbazine + Lomustine	0	1
Procarbazine + Lomustine	0	1

The categories are mutually exclusive. SJLIFE = St. Jude Lifetime cohort

Table S3. Pharmacokinetics of alkylating agents

Alkylating agent	Active/ prodrug	CYP3A4	CYP2B6	CYP2C19	Other	Polymorphism-altering pharmacokinetics
Cyclophosphamide	Prodrug	+	++	++	CYP3A5<br>GSTs	Yes(8, 9)
Ifosfamide	Prodrug	+	++	++	CYP2A6<br>CYP2B1<br>CYP2C8<br>CYP2C9<br>CYP3A5<br>GSTs	Yes (8, 10)
Procarbazine	Prodrug		+		CYP1A4<br>CYP3A5	Unknown
Busulfan	Active		+		CYP2C9	Yes (11, 12)
Melphalan	Active				-	No(13)
CCNU/Lomustine	Active				unknown	unknown
BCNU/Carmustine	Active				unknown	unknown
Chlorambucil	Active				GSTs	Yes (14)
Thiotepa	Prodrug		++		CYP3A4<br>CYP3A5	Yes(10)
Mechlorethamine	Active				unknown	unknown

GSTs = Glutathione S-transferases

Table S4. Results linear regression based on log-transformed AMH and interaction for Discovery cohort PanCareLIFE

Gene	Variant	Star-allele	Model	Variant, interaction	*N* (0/1/2) ‡	Beta (SE)	*P*-value
CYP2C19	rs4244285	*2	1	rs4244285	536/189/18	-0.019 (0.047)	0.692
				CED: 0		0 (ref) †	1.83E-28^
				>0 – 4000		-0.031 (0.063)	0.629
				≥ 4000-8000		-0.240 (0.072)	0.001
				≥ 8000		-0.727 (0.065)	5.83E-27
			2	rs4244285		0.025 (0.081)	0.756
				CED: 0		0 (ref) †	2.58E-21^
				>0 – 4000		0.001 (0.073)	0.986
				≥ 4000-8000		-0.227 (0.082)	0.006
				≥ 8000		-0.719 (0.076)	3.07E-20
				SNP*CED: 0	200/60/6	0 (ref) †	0.857^
				>0 – 4000	129/50/4	-0.107 (0.124)	0.386
				≥ 4000-8000	89/25/4	-0.051 (0.141)	0.718
				≥ 8000	118/54/4	-0.034 (0.124)	0.784
CYP2C19	rs12248560	*17	1	rs12248560	432/274/37	-0.017 (0.041)	0.674
				CED: 0		0 (ref) †	1.15E-28^
				>0 – 4000		-0.030 (0.063)	0.631
				≥ 4000-8000		-0.240 (0.072)	0.0009
				≥ 8000		-0.729 (0.065)	3.63E-27
			2	rs12248560		0.062 (0.068)	0.366
				CED: 0		0 (ref) †	3.06E-14^
				>0 – 4000		0.007 (0.082)	0.934
				≥ 4000-8000		-0.222 (0.092)	0.016
				≥ 8000		-0.620 (0.081)	5.88E-14
				SNP*CED: 0	161/92/13	0 (ref) †	0.150^
				>0 – 4000	99/77/7	-0.056 (0.108)	0.605
				≥ 4000-8000	67/44/7	-0.047 (0.119)	0.691
				≥ 8000	105/61/10	-0.240 (0.107)	**0.025**
CYP3A4	rs2740574	*1B	1	rs2740574	690/53/0	-0.004 (0.093)	0.963
				CED: 0		0 (ref) †	1.26E-28^
				>0 – 4000		-0.031 (0.063)	0.619

				≥ 4000-8000		-0.240 (0.072)	0.001
				≥ 8000		-0.729 (0.065)	3.68E-27
			2	rs2740574		-0.049 (0.152)	0.748
				CED: 0		0 (ref) †	1.46E-25^
				>0 – 4000		-0.046 (0.066)	0.487
				≥ 4000-8000		-0.259 (0.074)	0.0005
				≥ 8000		-0.714 (0.067)	1.11E-24
				SNP*CED: 0	246/20/0	0 (ref) †	0.243^
				>0 – 4000	165/18/0	0.166 (0.222)	0.455
				≥ 4000-8000	114/4/0	0.520 (0.364)	0.154
				≥ 8000	165/11/0	-0.202 (0.251)	0.420
CYP3A4	rs4986910	*3	1	rs4986910	735/8/0	-0.625 (0.252)	**0.013**
				CED: 0		0 (ref) †	6.51E-29^
				>0 – 4000		-0.027 (0.063)	0.672
				≥ 4000-8000		-0.234 (0.072)	0.001
				≥ 8000		-0.728 (0.065)	2.69E-27
			2	rs4986910		0.185 (0.515)	0.719
				CED: 0		0 (ref) †	9.83E-28^
				>0 – 4000		-0.027 (0.063)	0.663
				≥ 4000-8000		-0.215 (0.072)	0.003
				≥ 8000		-0.712 (0.064)	2.71E-26
				SNP*CED: 0	264/2/0	0 (ref) †	0.015^
				>0 – 4000	180/3/0	-0.317 (0.655)	0.629
				≥ 4000-8000	116/2/0	-1.558 (0.740)	**0.035**
				≥ 8000	175/1/0	-2.195 (0.821)	**0.008**
CYP3A4	rs35599367	*22	1	rs35599367	678/62/3	-0.001 (0.080)	0.988
				CED: 0		0 (ref) †	1.23E-28^
				>0 – 4000		-0.031 (0.063)	0.620
				≥ 4000-8000		-0.240 (0.072)	0.001
				≥ 8000		-0.729 (0.065)	3.75E-27
			2	rs35599367		0.006 (0.131)	0.966
				CED: 0		0 (ref) †	4.79E-28^
				>0 – 4000		-0.012 (0.066)	0.852
				≥ 4000-8000		-0.244 (0.076)	0.001

				≥ 8000		-0.740 (0.067)	5.23E-26
				SNP*CED: 0	241/24/1	0 (ref) †	0.465^
				>0 – 4000	169/14/0	-0.244 (0.223)	0.274
				≥ 4000-8000	106/11/1	0.038 (0.219)	0.861
				≥ 8000	162/13/1	0.137 (0.210)	0.515
CYP2B6	rs8192709	*2	1	rs8192709	678/63/2	0.047 (0.081)	0.560
				CED: 0		0 (ref) †	1.69E-28^
				> 0 – 4000		-0.030 (0.063)	0.637
				≥ 4000-8000		-0.238 (0.072)	0.001
				≥ 8000		-0.727 (0.065)	5.59E-27
			2	rs8192709		-0.020 (0.116)	0.860
				CED: 0		0 (ref) †	3.95E-29^
				> 0 – 4000		-0.037 (0.066)	0.579
				≥ 4000-8000		-0.229 (0.075)	0.002
				≥ 8000		-0.765 (0.067)	1.50E-27
				SNP*CED: 0	237/27/2	0 (ref) †	0.093^
				> 0 – 4000	167/16/0	0.038 (0.206)	0.855
				≥ 4000-8000	110/8/0	-0.209 (0.263)	0.428
				≥ 8000	164/12/0	0.489 (0.227)	**0.031**
CYP2B6	rs2279343	*6	1	rs2279343	410/279/54	-0.038 (0.039)	0.327
				CED: 0		0 (ref) †	1.11E-28^
				>0 – 4000		-0.033 (0.063)	0.603
				≥ 4000-8000		-0.238 (0.072)	0.001
				≥ 8000		-0.729 (0.065)	3.09E-27
			2	rs2279343		-0.077 (0.064)	0.225
				CED: 0		0 (ref) †	1.68E-17^
				>0 – 4000		-0.091 (0.081)	0.266
				≥ 4000-8000		-0.268 (0.098)	0.006
				≥ 8000		-0.738 (0.084)	8.02E-18
				SNP*CED: 0	147/98/21	0 (ref) †	0.696^
				>0 – 4000	106/67/10	0.118 (0.104)	0.256
				≥ 4000-8000	58/50/10	0.057 (0.115)	0.621
				≥ 8000	99/64/13	0.014 (0.102)	0.891
CYP2B6	rs3745274	*9	1	rs3745274	426/269/48	-0.045 (0.039)	0.250

				CED: 0		0 (ref) †	1.21E-28^
				>0 – 4000		-0.033 (0.063)	0.599
				≥ 4000-8000		-0.236 (0.072)	0.001
				≥ 8000		-0.729 (0.065)	3.22E-27
			2	rs3745274		-0.083 (0.064)	0.197
				CED: 0		0 (ref) †	7.03E-18^
				> 0 – 4000		-0.096 (0.079)	0.229
				≥ 4000-8000		-0.260 (0.096)	0.007
				≥ 8000		-0.730 (0.081)	2.77E-18
				SNP*CED: 0	154/94/18	0 (ref) †	0.562^
				> 0 – 4000	111/64/8	0.138 (0.105)	0.188
				≥ 4000-8000	58/51/9	0.047 (0.114)	0.679
				≥ 8000	103/60/13	0.001 (0.101)	0.991
CYP2B6	rs4802101	*1G	1	rs4802101	118/336/289	-0.006 (0.034)	0.857
				CED: 0		0 (ref) †	1.26E-28^
				> 0 – 4000		-0.032 (0.063)	0.616
				≥ 4000-8000		-0.240 (0.072)	0.001
				≥ 8000		-0.729 (0.065)	3.67E-27
			2	rs4802101		-0.083 (0.056)	0.142
				CED: 0		0 (ref) †	4.84E-11^
				> 0 – 4000		-0.183 (0.123)	0.139
				≥ 4000-8000		-0.346 (0.158)	0.029
				≥ 8000		-0.894 (0.126)	3.54E-12
				SNP*CED: 0	43/118/105	0 (ref) †	0.383^
				> 0 – 4000	32/88/63	0.125 (0.089)	0.160
				≥ 4000-8000	11/63/44	0.085 (0.112)	0.445
				≥ 8000	32/67/77	0.133 (0.087)	0.127

CED = Cyclophosphamide equivalent dose; AMH = Anti-Müllerian hormone

†reference is corresponding rs*CED 0 (ref). Multivariable model adjusted for 10 principal components, CED score and age at serum sampling

‡ *N*= alternative allele frequency is reported as 0/1/2 (recalculated based on allelic dosage), other analysis are performed with allelic dosage.

^ the reported *P*-value is the overall *P*-value for the analysis.

Table S5. Results logistic regression based on lowest tertile of AMH vs highest tertile of AMH (*N*=243 vs *N*= 240) and interaction in Discovery cohort PanCareLIFE

Gene	Variant	Star-allele	Model	Variant, interaction	*N* low AMH (0/1/2) ‡	*N* high AMH (0/1/2) ‡	**OR (95% CI)**	*P*-value
CYP2C19	rs4244285	*2	1	rs4244285	173/60/7	174/61/8	0.900 (0.620 - 1.308)	0.582
				CED: 0			1 (ref)	3.69E-14
				>0 – 4000			1.303 (0.774 - 2.193)	0.320
				≥ 4000-8000			4.311 (2.411 - 7.706)	8.24E-7
				≥ 8000			7.439 (4.287 - 12.909)	9.61E-13
			2	rs4244285			0.607 (0.287 - 1.283)	0.191
				CED: 0			1 (ref)	4.49E-11
				>0 – 4000			0.964 (0.525 - 1.769)	0.906
				≥ 4000-8000			4.268 (2.211 – 8.237)	0.00002
				≥ 8000			6.625 (3.510 - 12.504)	5.39E-9
				SNP*CED: 0	40/10/0	81/26/4	1 (ref)	0.240
				>0 – 4000	25/13/2	52/18/1	2.729 (0.959 – 7.767)	0.060
				≥ 4000-8000	41/9/2	20/7/2	1.104 (0.364 – 3.344)	0.861
				≥ 8000	67/28/3	21/10/1	1.557 (0.543 - 4.461)	0.410
CYP2C19	rs12248560	*17	1	rs12248560	147/74/19	145/90/8	1.041 (0.749 - 1.449)	0.809
				CED: 0			1 (ref)	4.27E-14
				>0 – 4000			1.291 (0.767 - 2.172)	0.336
				≥ 4000-8000			4.287 (2.398 - 7.663)	9.01E-7
				≥ 8000			7.325 (4.232 - 12.677)	1.12E-12
			2	rs12248560			0.888 (0.480 - 1.643)	0.705
				CED: 0			1 (ref)	4.52E-8
				>0 – 4000			1.250 (0.643 - 2.429)	0.511
				≥ 4000-8000			4.858 (2.337 – 10.100)	0.00002
				≥ 8000			5.367 (2.781 - 10.357)	5.47E-7
				SNP*CED: 0	34/13/3	68/40/3	1 (ref)	0.296
				>0 – 4000	24/14/2	40/29/2	1.106 (0.433 – 2.827)	0.834
				≥ 4000-8000	32/16/4	15/11/3	0.833 (0.331 – 2.099)	0.699
				≥ 8000	57/31/10	22/10/0	2.161 (0.817 – 5.717)	0.121

CYP3A4	rs2740574	*1B	1	rs2740574	225/15/0	226/17/0	1.105 (0.499 – 2.449)	0.805
				CED: 0			1 (ref)	4.22E-7
				>0 – 4000			1.288 (0.765 - 2.169)	0.341
				≥ 4000-8000			4.314 (2.413 - 7.715)	8.23E-7
				≥ 8000			7.352 (4.247 - 12.727)	1.04E-12
			2	rs2740574			2.104 (0.604 - 7.337)	0.243
				CED: 0			1 (ref)	1.53E-13
				>0 – 4000			1.425 (0.824 - 2.465)	0.204
				≥ 4000-8000			4.798 (2.629 - 8.754)	3.21E-7
				≥ 8000			7.532 (4.272 – 13.280)	2.99E-12
				SNP*CED: 0	45/5/0	105/6/0	1 (ref)	0.378
				>0 – 4000	37/3/0	63/8/0	0.314 (0.048 – 2.064)	0.228
				≥ 4000-8000	51/1/0	27/2/0	0.122 (0.008 – 1.976)	0.139
				≥ 8000	92/6/0	31/1/0	0.911 (0.075 – 11.135)	0.942
CYP3A4	rs4986910	*3	1	rs4986910	237/3/0	241/2/0	1.665 (0.182 – 15.234)	0.652
				CED: 0			1 (ref)	4.83E-14
				>0 – 4000			1.289 (0.766 - 2.170)	0.339
				≥ 4000-8000			4.263 (2.383 - 7.627)	0.000001
				≥ 8000			7.307 (4.223 - 12.644)	1.17E-12
			2	rs4986910			1.45E-10 (1.13E-40 - 1.87E+20)	0.522
				CED: 0			1 (ref)	9.62E-14
				>0 – 4000			1.187 (0.695 - 2.028)	0.531
				≥ 4000-8000			4.282 (2.375 - 7.723)	0.000001
				≥ 8000			7.074 (4.070 – 12.293)	3.96E-12
				SNP*CED: 0	50/0/0^	110/1/0	1 (ref)	0.784
				>0 – 4000	39/1/0	71/0/0		
				≥ 4000-8000	51/1/0	28/1/0		
				≥ 8000	97/1/0	32/0/0		
CYP3A4	rs35599367	*22	1	rs35599367	218/21/1	226/16/1	1.287 (0.660 – 2.512)	0.459
				CED: 0			1 (ref)	4.60E-14
				>0 – 4000			1.305 (0.775 - 2.197)	0.317
				≥ 4000-8000			4.236 (2.369 - 7.577)	0.000001
				≥ 8000			7.387 (4.265 - 12.795)	9.67E-13

			2	rs35599367			0.886 (0.279 - 2.815)	0.838
				CED: 0			1 (ref)	7.77E-14
				>0 – 4000			1.137 (0.661 - 1.957)	0.642
				≥ 4000-8000			4.187 (2.269 - 7.726)	0.000005
				≥ 8000			7.499 (4.239 – 13.265)	4.44E-12
				SNP*CED: 0	46/4/0	102/8/1	1 (ref)	0.183
				>0 – 4000	35/5/0	70/1/0	11.930 (0.994 – 143.151)	0.051
				≥ 4000-8000	45/6/1	25/4/0	1.351 (0.253 – 7.214)	0.725
				≥ 8000	92/6/0	29/3/0	0.678 (0.106 – 4.355)	0.682
CYP2B6	rs8192709	*2	1	rs8192709	218/22/0	220/23/0	1.245 (0.638 - 2.429)	0.521
				CED: 0			1 (ref)	3.54E-14
				0 – 4000			1.301 (0.773 - 2.190)	0.322
				4000-8000			4.339 (2.426 - 7.762)	7.55E-7
				≥ 8000			7.442 (4.290 - 12.909)	9.19E-13
			2	rs8192709			1.495 (0.546 - 4.091)	0.434
				CED: 0			1 (ref)	7.90E-14
				> 0 – 4000			1.311 (0.750 - 2.291)	0.341
				≥ 4000-8000			4.407 (2.388 – 8.131)	0.000002
				≥ 8000			8.147 (4.562 - 14.547)	1,33E-12
				SNP*CED: 0	43/7/0	99/12/0	1 (ref)	0.630
				> 0 – 4000	35/5/0	65/6/0	1.011 (0.197 - 5.194)	0.990
				≥ 4000-8000	47/5/0	27/2/0	0.994 (0.135 - 7.301)	0.995
				≥ 8000	93/5/0	29/3/0	0.327 (0.053 - 2.009)	0.227
CYP2B6	rs2279343	*6	1	rs2279343	130/90/20	138/82/23	1.071 (0.789 – 1.453)	0.661
				CED: 0			1 (ref)	3.82E-14
				>0 – 4000			1.299 (0.772 - 2.187)	0.325
				≥ 4000-8000			4.304 (2.408 - 7.690)	8.31E-7
				≥ 8000			7.373 (4.258 - 12.767)	9.86E-13
			2	rs2279343			1.287 (0.770 - 2.150)	0.336
				CED: 0			1 (ref)	1.81E-9
				>0 – 4000			1.590 (0.807 - 3.135)	0.180
				≥ 4000-8000			4.529 (2.102 - 9.758)	0.000115
				≥ 8000			8.945 (4.389 – 18.231)	1.63E-9

				SNP*CED: 0	23/22/5	62/38/11	1 (ref)	0.767
				>0 – 4000	24/13/3	41/24/6	0.692 (0.308 – 1.553)	0.372
				≥ 4000-8000	26/23/3	17/9/3	0.929 (0.374 - 2.311)	0.874
				≥ 8000	57/32/9	18/11/3	0.713 (0.319 - 1.593)	0.409
CYP2B6	rs3745274	*9	1	rs3745274	132/90/18	146/76/21	1.120 (0.825 – 1.520)	0.467
				CED: 0			1 (ref)	3.92E-14
				>0 – 4000			1.302 (0.774 - 2.193)	0.320
				≥ 4000-8000			4.295 (2.404 - 7.675)	8.61E-7
				≥ 8000			7.385 (4.264 - 12.788)	9.60E-13
			2	rs3745274			1.391 (0.828 - 2.337)	0.212
				CED: 0			1 (ref)	9.85E-10
				>0 – 4000			1.683 (0.865 - 3.275)	0.125
				≥ 4000-8000			4.407 (2.073 – 9.370)	0.000116
				≥ 8000			9.055 (4.511 – 18.174)	5.70E-10
				SNP*CED: 0	23/23/4	66/36/9	1 (ref)	0.589
				>0 – 4000	25/13/2	43/22/6	0.605 (0.267 – 1.374)	0.230
				≥ 4000-8000	25/24/3	18/8/3	0.974 (0.391 – 2.424)	0.955
				≥ 8000	59/30/9	19/10/3	0.689 (0.309 – 1.536)	0.363
CYP2B6	rs4802101	*1G	1	rs4802101	37/98/105	32/112/99	1.030 (0.778 – 1.365)	0.836
				CED: 0			1 (ref)	3.95E-14
				>0 – 4000			1.298 (0.771 - 2.187)	0.327
				≥ 4000-8000			4.306 (2.409 - 7.697)	8.31E-7
				≥ 8000			7.357 (4.248 - 12.740)	1.06E-12
			2	rs4802101			1.434 (0.859 - 2.394)	0.168
				CED: 0			1 (ref)	0.00001
				> 0 – 4000			2.319 (0.765 – 7.029)	0.137
				≥ 4000-8000			5.541 (1.505 – 20.402)	0.010
				≥ 8000			19.186 (5.811 – 63.344)	0.000001
				SNP*CED: 0	5/18/27	15/49/47	1 (ref)	0.301
				> 0 – 4000	6/21/13	12/32/27	0.644 (0.301 – 1.380)	0.258
				≥ 4000-8000	5/26/21	3/16/10	0.841 (0.344 – 2.055)	0.704
				≥ 8000	21/33/44	2/15/15	0.490 (0.228 – 1.053)	0.068

AMH = Anti-Müllerian Hormone; CED = cyclophosphamide equivalent dose; SNP = Single nucleotide polymorphisms; OR = Odds ratio; 95% CI = 95% Confidence interval

Multivariable model adjusted for 10 principal components and CED score.

‡ *N*= alternative allele frequency is reported as 0/1/2 (recalculated based on allelic dosage), other analysis are performed with allelic dosage.

^ There are no SNP carriers unexposed to CED for low AMH (controls). So it is not possible to run the interaction analyses for this SNP since there are no controls (low AMH) with the SNP but without CED.

Table S6a. Logistic regression based on low AMH (< -1.5SD) versus high AMH (≥ -1.5SD) and interaction in Discovery cohort PanCareLIFE

Gene	Variant	Star-allele	Model	Variant, interaction	*N* low AMH (0/1/2) ‡ (*N*=50)	*N* high AMH (0/1/2) ‡ (*N*=676)	**OR (95% CI)**	*P*-value
CYP3A4	rs4986910	*3	1	rs4986910	48/2/0	670/6/0	19.719 (2.12 – 183.410)	**0.009**
			2	rs4986910				
				SNP*CED: 0	5/0/0^	256/2/0		
				>0 – 4000	1/0/0	176/3/0		
				≥ 4000-8000	7/1/0	108/1/0		
				≥ 8000	35/1/0	130/0/0		
CYP2B6	rs8192709	*2	1	rs8192709	48/2/0	613/61/2	0.501 (0.111 – 2.250)	0.367
			2	rs8192709				
				SNP*CED: 0	5/0/0^	229/27/2		
				> 0 – 4000	1/0/0	163/16/0		
				≥ 4000-8000	7/1/0	102/7/0		
				≥ 8000	35/1/0	119/11/0		
CYP2C19	rs12248560	*17	1	rs12248560	31/15/4	389/254/33	1.053 (0.629 – 1.764)	0.844
			2	rs12248560				
				SNP*CED: 0	4/0/1	154/92/12	0.869 (0.180 – 4.191)	0.861
				>0 – 4000	0/1/0	97/75/7	4.578 (0.134 – 156.7)	0.399
				≥ 4000-8000	6/1/1	60/43/6	0.817 (0.107 – 6.252)	0.845
				≥ 8000	21/13/2	78/44/8	1.314 (0.243 – 7.116)	0.751

Low AMH < -1.5SD, high AMH: >-1.5SD; Significant p-values are reported in bold.

^There are no SNP carriers unexposed to CED for low AMH (controls). So it is not possible to run the interaction analyses for this SNP since there are no controls (low AMH) with the SNP but without CED.

‡ *N*= alternative allele frequency is reported as 0/1/2 (recalculated based on allelic dosage), other analysis are performed with allelic dosage.

AMH = Anti-Müllerian Hormone; CED = cyclophosphamide equivalent dose; SNP = Single nucleotide polymorphisms; OR = Odds ratio; 95% CI = 95% Confidence interval

Table S6b. Logistic regression based on low AMH (< -2SD) versus high AMH (≥ -2SD) and interaction in Discovery cohort PanCareLIFE

Gene	Variant	Star-allele	Model	Variant, interaction	*N* low AMH (0/1/2) ‡ (*N*=38)	*N* high AMH (0/1/2) ‡ (*N*=688)	**OR (95% CI)**	*P*-value
CYP3A4	rs4986910	*3	1	rs4986910	36/2/0	682/6/0	42.334 (4.01 – 446.95)	**0.002**
			2	rs4986910				
				SNP*CED: 0	3/0/0^	258/2/0		
				>0 – 4000	1/0/0	176/3/0		
				≥ 4000-8000	3/1/0	112/1/0		
				≥ 8000	29/1/0	136/0/0		
CYP2B6	rs8192709	*2	1	rs8192709	37/1/0	624/62/2	0.336 (0.042 - 2.665)	0.302
			2	rs8192709				
				SNP*CED: 0	3/0/0^	231/27/2		
				> 0 – 4000	1/0/0	163/16/0		
				≥ 4000-8000	4/0/0	105/8/0		
				≥ 8000	29/1/0	125/11/0		
CYP2C19	rs12248560	*17	1	rs12248560	23/12/3	397/257/34	1.189 (0.662 – 2.136)	0.562
			2	rs12248560				
				SNP*CED: 0	3/0/0^	155/92/13		
				>0 – 4000	0/1/0	97/75/7		
				≥ 4000-8000	3/0/1	63/44/6		
				≥ 8000	17/11/2	82/46/8		

Low AMH : < -2SD, high AMH: >-2SD; Significant p-values are reported in bold.

^ There are no SNP carriers unexposed to CED for low AMH (controls). So it is not possible to run the interaction analyses for this SNP since there are no controls (low AMH) with the SNP but without CED.

‡ *N*= alternative allele frequency is reported as 0/1/2 (recalculated based on allelic dosage), other analysis are performed with allelic dosage.

AMH = Anti-Müllerian Hormone; CED = cyclophosphamide equivalent dose; SNP = Single nucleotide polymorphisms; OR = Odds ratio; 95% CI = 95% Confidence interval

Table S7a. Sensitivity analysis: linear regression on Discovery cohort survivors receiving cyclophosphamide as part of the treatment-regimen

						Original Discovery cohort PanCareLIFE corrected for CED categories (*N*=743)			Only in cyclophosphamide group Discovery cohort PanCareLIFE corrected for CED categories (*N*=361) ^		
Gene	Variant	Ref> Alt	Mod el	Variant, interaction	CED *N* Cyclo *N* (0/1/2) ‡	Beta (SE)	95% CI	*P*-value	Beta (SE)	95% CI	*P*-value
CYP3A4	rs4986910	T>C	1	rs4986910	356/5/0	-0.625 (0.252)	-1.120 - -0.130	**0.013**	-1.031 (0.326)	-1.671 - -0.390	0.002
				CED: 0	288/4/0	0 (ref)		6.51E-29	-		
				>0 – 4000		-0.027 (0.063)	-0.150 - 0.097	0.672	0 (ref)		2.39E-14
				≥ 4000-8000		-0.234 (0.072)	-0.376 - -0.093	0.001	-0.145 (0.095)	-0.331 -0.041	0.127
				≥ 8000		-0.728 (0.065)	-0.854 - -0.601	2.69E-27	-0.739 (0.091)	-0.917 - -0.560	6.72E-15
			2	rs4986910		0.185 (0.515)	-0.826 - 1.197	0.719	-0.082 (0.432)	-0.932 -0.767	0.849
				CED: 0	CED	0 (ref)		9.83E-28	-		
				>0 – 4000	167/3/0	-0.027 (0.063)	-0.151 -0.096	0.663	0 (ref)		4.42E-14
				≥ 4000-8000	81/1/0	-0.215 (0.072)	-0.357 - -0.073	0.003	-0.105 (0.094)	-0.291 - 0.080	0.264
				≥ 8000	108/1/0	-0.712 (0.064)	-0.838 - -0.585	2.71E-26	-0.718 (0.090)	-0.895 - -0.541	2.14E-14
				SNP*CED: 0	cyclo	0 (ref)		0.015	-		
				>0 – 4000	167/3/0	-0.317 (0.655)	-1.603 – 0.969	0.629	0 (ref)		0.004
				≥ 4000-8000	48/0/0	-1.558 (0.740)	-3.010 - -0.107	**0.035**	-2.515 (0.818)	-4.124 - -0.907	0.002
				≥ 8000	73/1/0	-2.195 (0.821)	-3.806 - -0.584	**0.008**	-1.783 (0.809)	-3.375 - -0.191	0.028
CYP2B6	rs8192709	C>T	1	rs8192709	339/22/0	0.047 (0.081)	-0.111-0.205	0.560	-0.040 (0.159)	-0.354 -0.274	0.802
				CED: 0	273/19/0	0 (ref)		1.69E-28	-		
				> 0 – 4000		-0.030 (0.063)	-0.154-0.094	0.637	0 (ref)		6.03E-14
				≥ 4000-8000		-0.238 (0.072)	-0.380- -0.096	0.001	-0.148(0.096)	-0.337 - 0.041	0.124
				≥ 8000		-0.727 (0.065)	-0.854- -0.599	5.59E-27	-0.738 (0.092)	-0.919 - -0.557	1.62E-14
			2	rs8192709		-0.020 (0.116)	-0.247-0.207	0.860	-0.004 (0.205)	-0.406 - 0.399	0.985
				CED: 0	CED	0 (ref)		3.95E-29	-		
				> 0 – 4000	157/13/0	-0.037 (0.066)	-0.167-0.093	0.579	0 (ref)		1.03E-13
				≥ 4000-8000	78/4/0	-0.229 (0.075)	-0.376--0.082	0.002	-0.126 (0.099)	-0.321 - 0.069	0.206
				≥ 8000	104/5/0	-0.765 (0.067)	-0.897--0.632	1.50E-27	-0.744 (0.094)	-0.930 - -0.558	4.44E-14
				SNP*CED: 0	cyclo	0 (ref)		0.093	-		
				> 0 – 4000	157/13/0	0.038 (0.206)	-0.367-0.442	0.855	0 (ref)		0.417
				≥ 4000-8000	45/3/0	-0.209 (0.263)	-0.726-0.308	0.428	-0.424 (0.419)	-1.248 - 0.399	0.312

				≥ 8000	71/3/0	0.489 (0.227)	0.044-0.935	**0.031**	0.226 (0.387)	-0.536 - 0.988	0.561
CYP2C19	rs12248560	C>T	1	rs12248560	191/155/15	-0.017 (0.041)	-0.097 -0.063	0.674	-0.031 (0.064)	-0.158 -0.095	0.627
				CED: 0	158/123/11	0 (ref)		1.15E-28	-		
				>0 – 4000		-0.030 (0.063)	-0.155 -0.094	0.631	0 (ref)		6.70E-14
				≥ 4000-8000		-0.240 (0.072)	-0.382 - -0.098	0.0009	-0.146 (0.096)	-0.335 -0.042	0.128
				≥ 8000		-0.729 (0.065)	-0.856 - -0.601	3.63E-27	-0.736 (0.092)	-0.917 - -0.555	1.81E-14
			2	rs12248560		0.062 (0.068)	-0.072 -0.196	0.366	-0.0004 (0.093)	-0.183 -0.184	0.997
				CED: 0	CED	0 (ref)		3.06E-14	-		
				> 0 – 4000	93/70/7	0.007 (0.082)	-0.167 -0.154	0.934	0 (ref)		0.000002
				≥ 4000-8000	41/38/3	-0.222 (0.092)	-0.402 - -0.042	0.016	-0.219 (0.129)	-0.472 -0.035	0.090
				≥ 8000	57/47/5	-0.620 (0.081)	-0.778 - -0.461	5.88E-14	-0.632 (0.120)	-0.869 - -0.396	2.48E-7
				SNP*CED: 0	cyclo	0 (ref)		0.150			
				> 0 – 4000	93/70/7	-0.056 (0.108)	-0.268 -0.156	0.605	0 (ref)		0.163
				≥ 4000-8000	24/22/2	-0.047 (0.119)	-0.281 -0.187	0.691	0.130 (0.166)	-0.197 - 0.456	0.435
				≥ 8000	41/31/2	-0.240 (0.107)	-0.450 - -0.030	**0.025**	-0.201 (0.151)	-0.497 - 0.095	0.183

CED = cyclophosphamide equivalent dose; cyclo = cyclophosphamide; SNP = Single nucleotide polymorphisms. When the analysis is performed in the cyclophosphamide group, there is no CED=0 group. Thus the reference group is the 0-4000 group. Significant p-values are reported in bold.

‡ *N*= alternative allele frequency is reported as 0/1/2 (recalculated based on allelic dosage), other analysis are performed with allelic dosage.

^ In this group only patients who received cyclophosphamide as part of the treatment regimen are included in the analysis. Notably some patients receiving other alkylating agents such as Ifosfamide monotherapy are excluded.

Table S7b. Sensitivity analysis: linear regression on Discovery cohort survivors receiving cyclophosphamide monotherapy group

						original Discovery cohort PanCareLIFE corrected for CED categories (*N*=743)			Only in cyclophosphamide group Discovery cohort PanCareLIFE corrected for CED categories (*N*=250)		
Gene	Variant	Ref> Alt	Model	Variant, interaction	CED *N* Cyclo *N* (0/1/2) ‡	Beta (SE)	95% CI	*P*-value	Beta (SE)	95% CI	*P*-value
CYP3A4	rs4986910	T>C	1	rs4986910	247/3/0	-0.625 (0.252)	-1.120 - -0.130	**0.013**	-0.130 (0.240)	-0.603 - 0.343	0.588
				CED: 0	247/3/0	0 (ref)		6.51E-29	-		
				>0 – 4000		-0.027 (0.063)	-0.150 -0.097	0.672	0 (ref)		0.104
				≥ 4000-8000		-0.234 (0.072)	-0.376 - -0.093	0.001	-0.046 (0.065)	-0.175 - 0.083	0.483
				≥ 8000		-0.728 (0.065)	-0.854 - -0.601	2.69E-27	-0.157 (0.074)	-0.303 - -0.012	0.035
			2	rs4986910		0.185 (0.515)	-0.826 - 1.197	0.719	-0.138 (0.244)	-0.618 - 0.341	0.570
				CED: 0	CED^	0 (ref)		9.83E-28	-		
				>0 – 4000	162/3/0	-0.027 (0.063)	-0.151 -0.096	0.663	0 (ref)		0.104
				≥ 4000-8000	45/0/0	-0.215 (0.072)	-0.357 - -0.073	0.003	-0.044 (0.067)	-0.177 - 0.089	0.513
				≥ 8000	40/0/0	-0.712 (0.064)	-0.838 - -0.585	2.71E-26	-0.160 (0.075)	-0.308 - -0.012	0.034
				SNP*CED: 0	Cyclo^^	0 (ref)		0.015	-		
				>0 – 4000	162/3/0	-0.317 (0.655)	-1.603 – 0.969	0.629	0 (ref)		0.959
				≥ 4000-8000	45/0/0	-1.558 (0.740)	-3.010 - -0.107	**0.035**	-0.903 (8.473)	-17.596 - 15.791	0.915
				≥ 8000	40/0/0	-2.195 (0.821)	-3.806 - -0.584	**0.008**	0.544 (2.025)	-3.445 - 4.534	0.788
CYP2B6	rs8192709	C>T	1	rs8192709	233/17/0	0.047 (0.081)	-0.111-0.205	0.560	-0.085(0.098)	-0.278 - 0.107	0.385
				CED: 0	233/17/0	0 (ref)		1.69E-28	-		
				> 0 – 4000		-0.030 (0.063)	-0.154-0.094	0.637	0 (ref)		0.107
				≥ 4000-8000		-0.238 (0.072)	-0.380- -0.096	0.001	-0.045 (0.065)	-0.173 - 0.084	0.493
				≥ 8000		-0.727 (0.065)	-0.854- -0.599	5.59E-27	-0.157 (0.074)	-0.302 - -0.011	0.035
			2	rs8192709		-0.020 (0.116)	-0.247-0.207	0.860	-0.016 (0.117)	-0.245 - 0.214	0.893
				CED: 0	CED^	0 (ref)		3.95E-29	-		
				> 0 – 4000	153/12/0	-0.037 (0.066)	-0.167-0.093	0.579	0 (ref)		0.122
				≥ 4000-8000	42/3/0	-0.229 (0.075)	-0.376--0.082	0.002	-0.011 (0.068)	-0.145 - 0.123	0.874
				≥ 8000	38/2/0	-0.765 (0.067)	-0.897--0.632	1.50E-27	-0.154 (0.076)	-0.304 - -0.004	0.044
				SNP*CED: 0	Cyclo^^	0 (ref)		0.093	-		
				> 0 – 4000	153/12/0	0.038 (0.206)	-0.367-0.442	0.855	0 (ref)		0.170

				≥ 4000-8000	42/3/0	-0.209 (0.263)	-0.726-0.308	0.428	-0.467 (0.264)	-0.987 - 0.053	0.078
				≥ 8000	38/2/0	0.489 (0.227)	0.044-0.935	**0.031**	0.097 (0.302)	-0.498 - 0.691	0.748
CYP2C19	rs12248560	C>T	1	rs12248560	137/103/10	-0.017 (0.041)	-0.097 -0.063	0.674	0.026 (0.042)	-0.057 - 0.109	0.541
				CED: 0	137/103/10	0 (ref)		1.15E-28	-		
				>0 – 4000		-0.030 (0.063)	-0.155 -0.094	0.631	0 (ref)		0.107
				≥ 4000-8000		-0.240 (0.072)	-0.382 - -0.098	0.0009	-0.047 (0.065)	-0.176 - 0.082	0.473
				≥ 8000		-0.729 (0.065)	-0.856 - -0.601	3.63E-27	-0.156 (0.074)	-0.302 - -0.011	0.036
			2	rs12248560		0.062 (0.068)	-0.072 -0.196	0.366	0.005 (0.052)	-0.097 - 0.108	0.920
				CED: 0	CED^	0 (ref)		3.06E-14	-		
				> 0 – 4000	93/65/7	0.007 (0.082)	-0.167 -0.154	0.934	0 (ref)		0.126
				≥ 4000-8000	21/22/2	-0.222 (0.092)	-0.402 - -0.042	0.016	-0.082 (0.091)	-0.262 - 0.097	0.367
				≥ 8000	23/16/1	-0.620 (0.081)	-0.778 - -0.461	5.88E-14	-0.183 (0.092)	-0.365 - -0.001	0.049
				SNP*CED: 0	Cyclo^^	0 (ref)		0.150	-		
				> 0 – 4000	93/65/7	-0.056 (0.108)	-0.268 -0.156	0.605	0 (ref)		0.791
				≥ 4000-8000	21/22/2	-0.047 (0.119)	-0.281 -0.187	0.691	0.065 (0.113)	-0.157 - 0.287	0.566
				≥ 8000	23/16/1	-0.240 (0.107)	-0.450 - -0.030	**0.025**	0.059 (0.124)	-0.185 - 0.304	0.633

Analysis on the subgroup of survivors of the Discovery cohort who received cyclophosphamide monotherapy. Monotherapy refers to alkylating agents only. Notably these survivors may have received other non-alkylating agents during treatment. CED = cyclophosphamide equivalent dose; cyclo = cyclophosphamide; SNP = Single nucleotide polymorphisms

Significant p-values are reported in bold.

‡ *N*= alternative allele frequency is reported as 0/1/2 (recalculated based on allelic dosage), other analysis are performed with allelic dosage.

^ number of survivors with 0/1/2 alternative alleles per CED category

^^ number of survivors with 0/1/2 alternative alleles per cyclophosphamide category

Table S8. Sensitivity analysis: linear regression on Discovery cohort survivors with correction for age at diagnosis

Gene	Variant	Star-allele	Model	Variant, interaction	*N* (0/1/2) ‡	Discovery cohort PanCareLIFE Beta (SE)	Discovery cohort PanCareLIFE *P*-value	Discovery cohort PanCareLIFE correction for age at diagnosis Beta (SE)	Discovery cohort PanCareLIFE correction for age at diagnosis *P*-value
CYP2C19	rs4244285	*2	1	rs4244285	536/189/18	-0.019 (0.047)	0.692	-0.019 (0.047)	0.679
				Age at diagnosis		-	-	-0.012 (0.005)	0.013
				CED:0		0 (ref)	1.83E-28	0 (ref)	2.67E-27
				>0 – 4000		-0.031 (0.063)	0.629	-0.033 (0.063)	0.596
				≥ 4000-8000		-0.240 (0.072)	0.001	-0.208 (0.073)	0.005
				≥ 8000		-0.727 (0.065)	5.83E-27	-0.713 (0.065)	4.58E-26
			2	rs4244285		0.025 (0.081)	0.756	0.026 (0.081)	0.751
				CED: 0		0 (ref)	2.58E-21	0 (ref)	2.33E-20
				>0 – 4000		0.001 (0.073)	0.986	0.0001 (0.073)	0.998
				≥ 4000-8000		-0.227 (0.082)	0.006	-0.196 (0.082)	0.017
				≥ 8000		-0.719 (0.076)	3.07E-20	-0.703 (0.076)	1.77E-19
				SNP*CED: 0	200/60/6	0 (ref)	0.857	0 (ref)	0.838
				>0 – 4000	129/50/4	-0.107 (0.124)	0.386	-0.113 (0.124)	0.360
				≥ 4000-8000	89/25/4	-0.051 (0.141)	0.718	-0.042 (0.140)	0.766
				≥ 8000	118/54/4	-0.034 (0.124)	0.784	-0.039 (0.124)	0.750
CYP2C19	rs12248560	*17	1	rs12248560	432/274/37	-0.017 (0.041)	0.674	-0.021 (0.041)	0.612
				Age at diagnosis		-	-	-0.012 (0.005)	0.013
				CED: 0		0 (ref)	1.15E-28	0 (ref)	1.63E-27
				>0 – 4000		-0.030 (0.063)	0.631	-0.033 (0.063)	0.599
				≥ 4000-8000		-0.240 (0.072)	0.0009	-0.207 (0.073)	0.005
				≥ 8000		-0.729 (0.065)	3.63E-27	-0.715 (0.065)	2.86E-26
			2	rs12248560		0.062 (0.068)	0.366	0.060 (0.068)	0.376
				CED: 0		0 (ref)	3.06E-14	0 (ref)	8.81E-14
				>0 – 4000		0.007 (0.082)	0.934	-0.004 (0.082)	0.959
				≥ 4000-8000		-0.222 (0.092)	0.016	-0.188 (0.092)	0.042
				≥ 8000		-0.620 (0.081)	5.88E-14	-0.608 (0.081)	1.48E-13
				SNP*CED: 0	161/92/13	0 (ref)	0.150	0 (ref)	0.168
				>0 – 4000	99/77/7	-0.056 (0.108)	0.605	-0.067 (0.108)	0.537

				≥ 4000-8000	67/44/7	-0.047 (0.119)	0.691	-0.051 (0.119)	0.669
				≥ 8000	105/61/10	-0.240 (0.107)	**0.025**	-0.235 (0.107)	**0.028**
CYP3A4	rs2740574	*1B	1	rs2740574	690/53/0	-0.004 (0.093)	0.963	0.025 (0.093)	0.788
				Age at diagnosis		-	-	-0.012 (0.005)	0.013
				CED: 0		0 (ref)	1.26E-28	0 (ref)	1.90E-27
				>0 – 4000		-0.031 (0.063)	0.619	-0.035 (0.063)	0.579
				≥ 4000-8000		-0.240 (0.072)	0.001	-0.206 (0.073)	0.005
				≥ 8000		-0.729 (0.065)	3.68E-27	-0.715 (0.065)	2.95E-26
			2	rs2740574		-0.049 (0.152)	0.748	-0.031 (0.152)	0.836
				CED: 0		0 (ref)	1.46E-25	0 (ref)	1.17E-24
				>0 – 4000		-0.046 (0.066)	0.487	-0.049 (0.066)	0.458
				≥ 4000-8000		-0.259 (0.074)	0.0005	-0.226 (0.075)	0.003
				≥ 8000		-0.714 (0.067)	1.11E-24	-0.703 (0.067)	4.92E-24
				SNP*CED: 0	246/20/0	0 (ref)	0.243	0 (ref)	0.312
				>0 – 4000	165/18/0	0.166 (0.222)	0.455	0.163 (0.222)	0.464
				≥ 4000-8000	114/4/0	0.520 (0.364)	0.154	0.489 (0.363)	0.179
				≥ 8000	165/11/0	-0.202 (0.251)	0.420	-0.174 (0.250)	0.487
CYP3A4	rs4986910	*3	1	rs4986910	735/8/0	-0.625 (0.252)	**0.013**	-0.657(0.252)	**0.009**
				Age at diagnosis		-	-	-0.013 (0.005)	0.009
				CED: 0		0 (ref)	6.51E-29	0 (ref)	9.68E-28
				>0 – 4000		-0.027 (0.063)	0.672	-0.029 (0.063)	0.638
				≥ 4000-8000		-0.234 (0.072)	0.001	-0.200 (0.073)	0.006
				≥ 8000		-0.728 (0.065)	2.69E-27	-0.713 (0.064)	2.27E-26
			2	rs4986910		0.185 (0.515)	0.719	0.098 (0.515)	0.848
				CED: 0		0 (ref)	9.83E-28	0 (ref)	1.03E-26
				>0 – 4000		-0.027 (0.063)	0.663	-0.030 (0.063)	0.628
				≥ 4000-8000		-0.215 (0.072)	0.003	-0.184 (0.073)	0.012
				≥ 8000		-0.712 (0.064)	2.71E-26	-0.698 (0.064)	1.83E-25
				SNP*CED: 0	264/2/0	0 (ref)	0.015	0 (ref)	0.021
				>0 – 4000	180/3/0	-0.317 (0.655)	0.629	-0.270 (0.653)	0.680
				≥ 4000-8000	116/2/0	-1.558 (0.740)	**0.035**	-1.436 (0.739)	**0.052**
				≥ 8000	175/1/0	-2.195 (0.821)	**0.008**	-2.124 (0.818)	**0.010**
CYP3A4	rs35599367	*22	1	rs35599367	678/62/3	-0.001 (0.080)	0.988	-0.00001 (0.079)	0.9999

				Age at diagnosis		-	-	-0.012 (0.005)	0.013
				CED: 0		0 (ref)	1.23E-28	0 (ref)	1.77E-27
				>0 – 4000		-0.031 (0.063)	0.620	-0.034 (0.063)	0.587
				≥ 4000-8000		-0.240 (0.072)	0.001	-0.208 (0.073)	0.005
				≥ 8000		-0.729 (0.065)	3.75E-27	-0.715 (0.065)	2.95E-26
			2	rs35599367		0.006 (0.131)	0.966	-0.010 (0.130)	0.936
				CED: 0		0 (ref)	4.79E-28	0 (ref)	3.76E-27
				>0 – 4000		-0.012 (0.066)	0.852	-0.017 (0.066)	0.795
				≥ 4000-8000		-0.244 (0.076)	0.001	-0.213 (0.076)	0.005
				≥ 8000		-0.740 (0.067)	5.23E-26	-0.729 (0.067)	2.01E-25
				SNP*CED: 0	241/24/1	0 (ref)	0.465	0 (ref)	0.424
				>0 – 4000	169/14/0	-0.244 (0.223)	0.274	-0.225 (0.222)	0.312
				≥ 4000-8000	106/11/1	0.038 (0.219)	0.861	0.063 (0.219)	0.774
				≥ 8000	162/13/1	0.137 (0.210)	0.515	0.174 (0.209)	0.407
CYP2B6	rs8192709	*2	1	rs8192709	678/63/2	0.047 (0.081)	0.560	0.043 (0.080)	0.592
				Age at diagnosis		-	-	-0.012 (0.005)	0.014
				CED: 0		0 (ref)	1.69E-28	0 (ref)	2,28E-27
				> 0 – 4000		-0.030 (0.063)	0.637	-0.033 (0.063)	0.602
				≥ 4000-8000		-0.238 (0.072)	0.001	-0.206 (0.073)	0.005
				≥ 8000		-0.727 (0.065)	5.59E-27	-0.713 (0.065)	4.17E-26
			2	rs8192709		-0.020 (0.116)	0.860	-0.039 (0.115)	0.736
				CED: 0		0 (ref)	3.95E-29	0 (ref)	4.06E-28
				> 0 – 4000		-0.037 (0.066)	0.579	-0.045 (0.066)	0.498
				≥ 4000-8000		-0.229 (0.075)	0.002	-0.197 (0.076)	0.009
				≥ 8000		-0.765 (0.067)	1.50E-27	-0.753 (0.067)	7,55E-27
				SNP*CED: 0	237/27/2	0 (ref)	0.093	0 (ref)	0.084
				> 0 – 4000	167/16/0	0.038 (0.206)	0.855	0.087 (0.206)	0.674
				≥ 4000-8000	110/8/0	-0.209 (0.263)	0.428	-0.202 (0.262)	0.442
				≥ 8000	164/12/0	0.489 (0.227)	**0.031**	0.505 (0.226)	**0.026**
CYP2B6	rs2279343	*6	1	rs2279343	410/279/54	-0.038 (0.039)	0.327	-0.039 (0.039)	0.315
				Age at diagnosis		-	-	-0.012 (0.005)	0.013
				CED: 0		0 (ref)	1.11E-28	0 (ref)	1.55E-27
				>0 – 4000		-0.033 (0.063)	0.603	-0.036 (0.063)	0.569

				≥ 4000-8000		-0.238 (0.072)	0.001	-0.205 (0.073)	0.005
				≥ 8000		-0.729 (0.065)	3.09E-27	-0.716 (0.065)	2.43E-26
			2	rs2279343		-0.077 (0.064)	0.225	-0.075 (0.064)	0.239
				CED: 0		0 (ref)	1.68E-17	0 (ref)	1.56E-16
				>0 – 4000		-0.091 (0.081)	0.266	-0.091 (0.081)	0.262
				≥ 4000-8000		-0.268 (0.098)	0.006	-0.237 (0.098)	0.016
				≥ 8000		-0.738 (0.084)	8.02E-18	-0.719 (0.084)	5,21E-17
				SNP*CED: 0	147/98/21	0 (ref)	0.696	0 (ref)	0.697
				>0 – 4000	106/67/10	0.118 (0.104)	0.256	0.113 (0.103)	0.274
				≥ 4000-8000	58/50/10	0.057 (0.115)	0.621	0.058 (0.114)	0.610
				≥ 8000	99/64/13	0.014 (0.102)	0.891	0.004 (0.102)	0.969
CYP2B6	rs3745274	*9	1	rs3745274	426/269/48	-0.045 (0.039)	0.250	-0.046 (0.039)	0.237
				Age at diagnosis		-	-	-0.012 (0.005)	0.013
				CED: 0		0 (ref)	1.21E-28	0 (ref)	1.67E-27
				>0 – 4000		-0.033 (0.063)	0.599	-0.036 (0.063)	0.565
				≥ 4000-8000		-0.236 (0.072)	0.001	-0.203 (0.073)	0.006
				≥ 8000		-0.729 (0.065)	3.22E-27	-0.715 (0.065)	2.54E-26
			2	rs3745274		-0.083 (0.064)	0.197	-0.084 (0.064)	0.190
				CED: 0		0 (ref)	7.03E-18	0 (ref)	5.11E-17
				>0 – 4000		-0.096 (0.079)	0.229	-0.098 (0.079)	0.214
				≥ 4000-8000		-0.260 (0.096)	0.007	-0.231 (0.096)	0.017
				≥ 8000		-0.730 (0.081)	2.77E-18	-0.713 (0.081)	1.38E-17
				SNP*CED: 0	154/94/18	0 (ref)	0.562	0 (ref)	0.545
				>0 – 4000	111/64/8	0.138 (0.105)	0.188	0.138 (0.105)	0.188
				≥ 4000-8000	58/51/9	0.047 (0.114)	0.679	0.054 (0.114)	0.635
				≥ 8000	103/60/13	0.001 (0.101)	0.991	-0.004 (0.101)	0.970
CYP2B6	rs4802101	*1G	1	rs4802101	118/336/289	-0.006 (0.034)	0.857	-0.006 (0.034)	0.854
				Age at diagnosis		-	-	-0.012 (0.005)	0.013
				CED: 0		0 (ref)	1.26E-28	0 (ref)	1.79E-27
				>0 – 4000		-0.032 (0.063)	0.616	-0.035 (0.063)	0.582
				≥ 4000-8000		-0.240 (0.072)	0.001	-0.208 (0.073)	0.005
				≥ 8000		-0.729 (0.065)	3.67E-27	-0.715 (0.065)	2.87E-26
			2	rs4802101		-0.083 (0.056)	0.142	-0.078 (0.056)	0.165

	CED: 0		0 (ref)	4.84E-11	0 (ref)	1.34E-10
	> 0 – 4000		-0.183 (0.123)	0.139	-0.174 (0.123)	0.158
	≥ 4000-8000		-0.346 (0.158)	0.029	-0.306 (0.159)	0.054
	≥ 8000		-0.894 (0.126)	3.54E-12	-0.873 (0.126)	1.01E-11
	SNP*CED: 0	43/118/105	0 (ref)	0.383	0 (ref)	0.436
	> 0 – 4000	32/88/63	0.125 (0.089)	0.160	0.115 (0.089)	0.194
	≥ 4000-8000	11/63/44	0.085 (0.112)	0.445	0.079 (0.111)	0.478
	≥ 8000	32/67/77	0.133 (0.087)	0.127	0.127 (0.086)	0.143

SE = standard error. CED = cyclophosphamide equivalent dose; SNP = Single nucleotide polymorphisms; Significant p-values are reported in bold.

‡ *N*= alternative allele frequency is reported as 0/1/2 (recalculated based on allelic dosage), other analysis are performed with allelic dosage.

Table S9. Sensitivity analysis: linear regression on Discovery cohort survivors corrected for linear CED score and interaction with cyclophosphamide

			Discovery cohort PCL corrected for CED categories		Discovery cohort PCL corrected for CED linear		*N* (0/1/2) ‡	Interaction with cyclophosphamide categories, corrected for CED categories^		Interaction with cyclophosphamide linear, corrected for CED linear^^	
Gene	Model	Variant, interaction	Beta (SE)	*P*-value	Beta (SE)	*P*-value		Beta (SE)	*P*-value	Beta (SE)	*P*-value
CYP3A4*3	1	rs4986910	-0.625 (0.252)	**0.013**	-0.610 (0.276)	**0.027**	735/8/0	-0.625 (0.252)	**0.013**	-1.088 (0.335)	**0.001**
		CED: 0	0 (ref)	6.51E-29	-2.90E-8 (5.20E-8)	0.577		0 (ref)	6.51E-29	-2.79E-8 (5.26E-8)	0.596
		>0 – 4000	-0.027 (0.063)	0.672				-0.027 (0.063)	0.672		
		≥ 4000-8000	-0.234 (0.072)	0.001				-0.234 (0.072)	0.001		
		≥ 8000	-0.728 (0.065)	2.69E-27				-0.728 (0.065)	2.69E-27		
	2	rs4986910	0.185 (0.515)	0.719	-0.018 (0.324)	0.957		0.313 (0.449)	0.487	-0.419 (0.455)	0.357
		CED: 0	0 (ref)	9.83E-28	-2.80E-8 (5.17E-8)	0.588		0 (ref)		-2.74E-8 (5.25E-8)	0.602
		>0 – 4000	-0.027 (0.063)	0.663				-0.020 (0.063)	0.754		
		≥ 4000-8000	-0.215 (0.072)	0.003				-0.217 (0.072)	0.003		
		≥ 8000	-0.712 (0.064)	2.71E-26				-0.705 (0.064)	5.34E-26		
		SNP*CED:					SNP* cyclo				
		0	0 (ref)	0.015	-0.00008 (0.00002)	**0.001**	447/4/0	0 (ref)		-0.0001 (0.0001)	**0.031**
		>0 – 4000	-0.317 (0.655)	0.629			167/3/0	-1.085 (0.563)	0.054		
		≥ 4000-8000	-1.558 (0.740)	**0.035**			48/0/0	-20.893 (8.541)	**0.015**		
		≥ 8000	-2.195 (0.821)	**0.008**			73/1/0	-2.219 (0.780)	**0.005**		
CYP2B6*2	1	rs8192709	0.047 (0.081)	0.560	0.087 (0.088)	0.323	678/63/2	0.047 (0.081)	0.560	-0.039 (0.157)	0.805
		CED: 0	0 (ref)	1.69E-28	-2.76E-8 (5.22E-8)	0.597		0 (ref)	1.69E-28	-2.67E-8 (5.32E-8)	0.616
		> 0 – 4000	-0.030 (0.063)	0.637				-0.030 (0.063)	0.637		
		≥ 4000-8000	-0.238 (0.072)	0.001				-0.238 (0.072)	0.001		
		≥ 8000	-0.727 (0.065)	5.59E-27				-0.727 (0.065)	5.59E-27		
	2	rs8192709	-0.020 (0.116)	0.860	0.131 (0.091)	0.151		0.084 (0.097)	0.388	0.287 (0.186)	0.124
		CED: 0	0 (ref)	3.95E-29	-2.61E-8 (5.21E-8)	0.617		0 (ref)		-2.52E-8 (5.29E-8)	0.634
		> 0 – 4000	-0.037 (0.066)	0.579				-0.023 (0.065)	0.729		

		≥ 4000-8000	-0.229 (0.075)	0.002				-0.234 (0.073)	0.001		
		≥ 8000	-0.765 (0.067)	1.50E-27				-0.718 (0.066)	6.87E-26		
		SNP*CED:					SNP* cyclo				
		0	0 (ref)	0.093	-0.000006 (0.000003)	0.064	405/44/2	0 (ref)		-0.0001 (0.00002)	**0.001**
		> 0 – 4000	0.038 (0.206)	0.855			157/13/0	-0.084 (0.198)	0.672		
		≥ 4000-8000	-0.209 (0.263)	0.428			45/3/0	-0.069 (0.342)	0.839		
		≥ 8000	0.489 (0.227)	**0.031**			71/3/0	-0.350 (0.391)	0.370		
CYP2C19*17	1	rs12248560	-0.017 (0.041)	0.674	-0.011 (0.045)	0.804	432/274/37	-0.017 (0.041)	0.674	-0.082 (0.058)	0.157
		CED: 0	0 (ref)	1.15E-28	-2.90E-8 (5.22E-8)	0.579		0 (ref)	1.15E-28	-2.18E-8 (5.37E-8)	0.689
		>0 – 4000	-0.030 (0.063)	0.631				-0.030 (0.063)	0.631		
		≥ 4000-8000	-0.240 (0.072)	0.0009				-0.240 (0.072)	0.001		
		≥ 8000	-0.729 (0.065)	3.63E-27				-0.729 (0.065)	3.63E-27		
	2	rs12248560	0.062 (0.068)	0.366	-0.007 (0.045)	0.875		0.005 (0.054)	0.921	-0.085 (0.058)	0.146
		CED: 0	0 (ref)	3.06E-14	-1.95E-8 (5.31E-8)	0.714		0 (ref)		-2.67E-8 (5.46E-8)	0.624
		> 0 – 4000	0.007 (0.082)	0.934				0.0002 (0.073)	0.998		
		≥ 4000-8000	-0.222 (0.092)	0.016				-0.269 (0.078)	0.001		
		≥ 8000	-0.620 (0.081)	5.88E-14				-0.695 (0.071)	1.58E-21		
		SNP*CED:					SNP* cyclo				
		0	0 (ref)	0.150	-2.88E-7 (2.89E-7)	0.319	274/151/26	0 (ref)		1.60E-7 (3.09E-7)	0.604
		> 0 – 4000	-0.056 (0.108)	0.605			93/70/7	-0.066 (0.084)	0.431		
		≥ 4000-8000	-0.047 (0.119)	0.691			24/22/2	0.162 (0.118)	0.171		
		≥ 8000	-0.240 (0.107)	**0.025**			41/31/2	-0.200 (0.124)	0.108		

PCL = PanCareLIFE cohort; CED = cyclophosphamide equivalent dose; SNP = Single nucleotide polymorphisms; cyclo = cyclophosphamide; Significant p-values are reported in bold.

‡ *N*= alternative allele frequency is reported as 0/1/2 (recalculated based on allelic dosage), other analysis are performed with allelic dosage.

^ Multivariable model is adjusted for 10 principal components, CED score and age at serum sampling. For the interaction model cyclophosphamide categories are used instead of CED to evaluate the contribution of cyclophosphamide in the interaction. Thus all other alkylating agents contributing to the CED score are not used to create the categories in the interaction model.

^^ Multivariable model is adjusted for 10 principal components, CED linear and age at serum sampling. For the interaction model cyclophosphamide linear is used instead of CED linear to evaluate the contribution of cyclophosphamide in the interaction.

Table S10. Logistic regression lowest tertile AMH vs highest tertile per year of age

Gene	Variant	Star-allele	Model	Variant, interaction	*N* low AMH (0/1/2) ‡	*N* high AMH (0/1/2) ‡	**OR (95% CI)**	*P*-value
CYP3A4	rs4986910	*3	1	rs4986910	236	243	1.951 (0.199 – 19.178)	0.566
			2	rs4986910	233/3/0	241/2/0	0.098 (0.000004 – 2360.5)	0.652
				SNP*CED: 0	45/0/0	112/1/0		
				>0 – 4000	39/1/0	75/0/0	57926.5 (2.73E-41 – 9.223E+15)	0.837
				≥ 4000-8000	50/1/0	26/1/0	6.298 (0.0002 – 256814.8)	0.734
				≥ 8000	99/1/0	28/0/0	141.787 (0.000007 – 2790864397)	0.563
CYP2B6	rs8192709	*2	1	rs8192709	236	243	1.343 (0.671 – 2.686)	0.404
			2	rs8192709	215/21/0	22/21/0	1.151(0.373 – 3.556)	0.807
				SNP*CED: 0	40/5/0	102/11/0		
				> 0 – 4000	34/6/0	69/6/0	1.810 (0.342 – 9.580)	0.485
				≥ 4000-8000	45/6/0	25/2/0	1.423 (0.188 – 10.758)	0.732
				≥ 8000	96/4/0	26/2/0	0.475 (0.059 – 3.833)	0.484
CYP2C19	rs12248560	*17	1	rs12248560	236	243	0.987 (0.709 – 1.374)	0.938
			2	rs12248560	146/72/18	145/88/0	0.860 (0.466 – 1.590)	0.631
				SNP*CED: 0	31/11/3	69/40/4		
				>0 – 4000	23/15/2	43/29/3	1.200 (0.486 – 2.966)	0.692
				≥ 4000-8000	33/15/3	13/11/3	0.674 (0.260 – 1.749)	0.417
				≥ 8000	59/31/10	20/8/0	2.188 (0.810 – 5.907)	0.122

Low AMH: lowest tertile AMH per year of age; High AMH: highest tertile AMH per year of age; AMH = Anti-Müllerian Hormone; CED = cyclophosphamide equivalent dose; SNP = Single nucleotide polymorphisms; OR = Odds ratio; 95% CI = 95% Confidence interval; Significant p-values are reported in bold.
‡ *N*= alternative allele frequency is reported as 0/1/2 (recalculated based on allelic dosage), other analysis are performed with allelic dosage.

Table S11. Results of the replication analysis in the SJLIFE cohort

Gene	Variant	Model	Variant, interaction	*N* (0/1/2) ‡	Beta (SE)	*P*-value
CYP3A4*3	rs4986910	1	rs4986910	*N*=391<br>379/12/0	-0.88 (0.37)	0.02
			CED: 0	-	0 (ref)	0.13
			>0 – 4000	-	0.16 (0.29)	0.59
			≥ 4000-8000	-	-0.23 (0.17)	0.17
			≥ 8000	-	-0.31 (0.16)	0.05
		2	rs4986910	-	-0.81 (0.52)	0.12
			CED: 0	-	0 (ref)	0.15
			>0 – 4000	-	0.15 (0.30)	0.62
			≥ 4000-8000	-	-0.21 (0.17)	0.22
			≥ 8000	-	-0.32 (0.16)	0.05
			SNP*CED: 0	192/6/0	0 (ref)	0.82
			>0 – 4000	20/1/0	0.20 (1.38)	0.88
			≥ 4000-8000	74/4/0	-0.46 (0.84)	0.58
			≥ 8000	93/1/0	0.83 (1.36)	0.54
CYP2B6*2	rs8192709	1	rs8192709	*N*=390*<br>345/40/5	0.06 (0.18)	0.74
			CED: 0	-	0 (ref)	0.15
			>0 – 4000	-	0.15 (0.29)	0.62
			≥ 4000-8000	-	-0.25 (0.17)	0.14
			≥ 8000	-	-0.29 (0.16)	0.07
		2	rs8192709	-	-0.11 (0.29)	0.72
			CED: 0	-	0 (ref)	0.09
			> 0 – 4000	-	0.14 (0.31)	0.64
			≥ 4000-8000	-	-0.24 (0.18)	0.18
			≥ 8000	-	-0.39 (0.17)	0.02
			SNP*CED: 0	179/17/1 *	0 (ref)	0.44
			> 0 – 4000	19/2/0	-0.09 (0.98)	0.92
			≥ 4000-8000	67/7/4	0.01 (0.40)	0.98
			≥ 8000	80/14/0	0.69 (0.47)	0.14

CYP2C19*17	rs12248560	1	rs12248560	*N*=390*<br>241/131/18	-0.01 (0.11)	0.91
			CED: 0	-	0 (ref)	0.15
			>0 – 4000	-	0.13 (0.29)	0.65
			≥ 4000-8000	-	-0.25 (0.17)	0.14
			≥ 8000	-	-0.30 (0.16)	0.06
		2	rs12248560	-	-0.15 (0.16)	0.38
			CED: 0	-	0 (ref)	0.15
			>0 – 4000	-	-0.07 (0.34)	0.84
			≥ 4000-8000	-	-0.28 (0.21)	0.17
			≥ 8000	-	-0.44 (0.20)	0.03
			SNP*CED: 0	125/66/6*	0 (ref)	0.53
			>0 – 4000	15/5/1	0.58 (0.52)	0.26
			≥ 4000-8000	51/23/4	0.08 (0.29)	0.78
			≥ 8000	50/37/7	0.30 (0.26)	0.26

‡ *N*= alternative allele frequency is reported as 0/1/2 (recalculated based on allelic dosage), other analysis should be performed with allelic dosage.
* 1 missing genotype. SJLIFE = St. Jude Lifetime Cohort.
AMH = Anti-Müllerian Hormone; CED = cyclophosphamide equivalent dose; SNP = Single nucleotide polymorphisms; Significant p-values are reported in bold.

Table S12. CYP450 Pharmacogenetics

Gene + Star allele	Variant	Ref> Alt	POS (B38)(15)	Classification/ Variant type	Functionality	Metabolism of cyclophosphamide or alkylators (16, 17)
*CYP2C19 *2*	rs4244285	681G > A (C) (18)	10: 94781859	Splicing I331V defect	*2/*2 poor metaboliser(19)<br><br>No function(20)<br><br>Synonymous variant (15) cryptic splice acceptor activation, protein loss of function (21)<br><br>Defective allele(22)<br><br>Truncated and non-functional enzyme (23)	CYP2C19 enzyme is involved in metabolism of cyclophosphamide and ifosfamide. A splice site mutation in exon 5 (CYP2C19*2) is one of the most predominant null alleles. Poor metabolizers are expected to have a reduced response + low toxicity after cyclophosphamide treatment, due to decreased CYP2C19-mediated activation(19).<br><br>In a Japanese and European trial CYP2C19*2 had no effect on the pharmacokinetics of cyclophosphamide. Notably cyclophosphamide is activated via multiple CYP enzyme pathways.(19)<br><br>AA + breast cancer + cyclophosphamide, doxorubicin and fluorouracil (FAC): increased risk of neutropenia versus (vs) GG.(24, 25)<br><br>AA + Systemic Lupus Erythematosus + cyclophosphamide: decreased cyclophosphamide metabolism (lower concentrations of active metabolite + decreased risk of toxicity (ovarian, gastrointestinal, or hematological)) vs GG.(18, 24)<br><br>AA + Breast Cancer + cyclophosphamide and doxorubicin treatment: increased risk of poorer outcome vs GG.(24, 26)<br><br>CYP2C19*2 variants have a protective effect on ovarian function (27, 28).<br><br>There are limited data correlating genotype to cyclophosphamide pharmacokinetics (29)
*CYP2C19*17*	**rs12248560**	C>A / C>T -806C>T<br><br>4195C > T (18)	10: 94761900	Intron variant, benign (15)	novel allele carrying -806C>T and -3402C>T: effect on promoter	CC genotype + breast cancer + cyclophosphamide, doxorubicin and fluorouracil (FAC): increased risk for leukopenia compared to CT or TT genotype.(24, 25)

					activity; effect on RNA abundance(30)<br>increased function (20)<br>Ultra rapid metaboliser (22)	C>T transition in the promoter creates a consensus binding site for the GATA transcription factor family and results in increased CYP2C19 expression and activity(24)
*CYP3A4*1B*	rs2740574	-392A>G (31)<br>C>T (forward strand) (15)<br>C>T/A/G (32)<br><br>-290A>G (in gene promoter) (33)	7: 99784473	Benign, intergenic variant(15)<br>promotor poly-morphism (21)	Alt allele: increases expression: higher activity(34)<br>Increased transcription in vitro (35)<br>Decreased clearance(36)<br>decreased metabolism of cyclophosphamide (37)	The CYP3A subfamily is involved in metabolism of >50 % of clinically used drugs, including anticancer drugs (cyclophosphamide, ifosfamide, thiotepa, etoposide, teniposide, docetaxel, paclitaxel, irinotecan, toremifene, vinblastine, vincristine, vinorelbine, gefitinib, imatinib, and erlotinib). Enzyme activity ranges widely and is largely affected by non-genetic factors (age, health status, endogenous hormone levels, environmental stimuli). About 40 variants are described for CYP3A4, but genetic variability in CYP3A alone is insufficient to explain its widely ranging enzyme activity.(19) 30–60% of liver cytochrome P450 protein is CYP3A4(38). It has been shown that the CYP3A4*1B allele is linked to the CYP3A5*1 allele, and in this way is correlated with an increased total CYP3A activity, mainly caused by the CYP3A5 expressor phenotype (39-43).<br><br>Premenopausal + CC + breast cancer + cyclophosphamide: longer time before chemotherapy-induced ovarian failure vs TT.(24, 33)<br><br>Transcription regulation and functional significance are controversial (44, 45). In vitro: increased transcription/expression, leading theoretically in vivo to higher enzymatic activity, higher cyclophosphamide activation and higher risk of ovarian failure (35). (35, 38).<br><br>CYP3A4*1B + cyclophosphamide-based chemotherapy in young breast cancer survivors: higher risk of ovarian failure vs wildtype genotype(33)<br><br>CYP3A4*1B gives worse cancer survival vs wildtype (46).<br>Decreased CYP3A4 function in carriers of the CYP3A4*1B variant genotypes (47, 48).

						Notably mechanisms for cancer survival and ovarian failure are more complex than chemotherapy metabolism.<br><br>There are limited data correlating genotype to cyclophosphamide pharmacokinetics.(49, 50)<br><br>No impact of CYP3A4*1B genotypes on cyclophosphamide and 4-hydroxycyclophosphamide clearance (underpowered study) (49).
*CYP3A4*3*	**rs4986910**	A>G<br>23171T >C (31, 51)<br>T1437C (52)	7: 99760901	Missense Met445Thr	Clinical significance not reported. (21)<br>Possibly decreased? (34)<br>Unclear/not significant(53)	Allele G is not associated with metabolism of ambrisentan, aripiprazole, atorvastatin, donepezil or olanzapine in healthy individuals as compared to allele A. (24, 54)<br><br>CYP3A4*3 + *20 + *22 are associated with increased exposure to fentanyl, imatinib or quetiapine and with increased clearance of fesoterodine in healthy individuals as compared to CYP3A4*1. (24, 54)<br><br>Genotype AG is not associated with response to atorvastatin as compared to genotype AA.(24, 53)<br><br>No reported effect on metabolism of cyclophosphamide. (24)
*CYP3A4*22*	rs35599367	G>A	7: 99768693	Intron variant.(15)	Intron variant.(15)<br><br>Reduced mRNA expression, low enzyme activity(54)<br><br>Decrease of function, decrease of clearance(55)	The T allele was associated with decreased clearance of sunitinib .(56)<br><br>*CYP3A4*22* proves correlated with a lower CYP3A4 enzymatic activity in vivo in cancer patients, as determined with the golden standard probes Midazolam and the Erytromycin Breath test.(57)<br>*CYP3A4*22* is correlated with an increased toxicity on paclitaxel(58)<br>*CYP3A4*22* has a small but clinical insignificant impact on the pharmacokinetics of sunitinib.(19)<br><br>*CYP3A4 *22*: increased simvastatin concentrations and increased cholesterol-lowering response.(55)

						*CYP3A4 *22*: significantly decreased CYP3A4 enzyme level and activity and altered pharmacokinetics and dynamics of simvastatin, atorvastatin, and lovastatin.(59)<br><br>No information on alkylating agents or cyclophosphamide specifically.
*CYP2B6*2*	**rs8192709**	64C > T (18)	19: 40991369	Missense Arg22Cys	Normal function (60)<br><br>Missense Clinical significance not reported (21)	There are limited data correlating genotype to cyclophosphamide pharmacokinetics.(61, 62)<br><br>rs8192709: response to immunosuppressive drugs (P =.0167 for cyclophosphamide-induced hemorrhagic cystitis)(63)<br><br>Allele T + cyclophosphamide and doxorubicin + Breast Neoplasms: increased likelihood of dose delay vs allele C.(26)<br><br>CC genotype + leukemia + cyclophosphamide in recipients of HLA-identical hematopoietic stem cell transplantation: decreased, but not absent, risk for hemorrhagic cystitis vs CT or TT genotype.(64)
*CYP2B6*6*	rs2279343	785A>G (18, 21)	19: 41009358	Missense Lys262Arg	Decreased cyclophosphamide clearance<br><br>Increased risk mucositis (63) thus possibly also decreased clearance	CYP2B6 converts cyclophosphamide to active form 4-hydroxycyclophosphamide. CYP2B6*6 = most common functionally deficient allele. One CYP2B6*6 variant allele: lower cyclophosphamide clearance vs homozygous wildtype patients (no impact on clinical outcome). *6 allele: higher rate of cyclophosphamide 4-hydroxylation. (19)<br><br>Overall effect of CYP2B6*6 expression on pharmacokinetics + therapeutic efficacy/toxicity of cyclophosphamide is difficult to predict and depends on the dominant effect (reduced enzyme expression or increased specific enzyme activity). (19)<br><br>rs2279343: response to immunosuppressive drugs (P =0.46E-20 for cyclophosphamide-induced mucositis)(63)<br><br>*1/*1 genotype + chronic lymphocytic leukemia + combination cyclophosphamide and fludarabine treatment: higher chance of complete response and higher risk of drug toxicities vs *1/*6 or *6/*6 genotype.(9)

						*1/*1 diplotype + B-cell non-Hodgkin's lymphoma: increased cyclophosphamide clearance vs *1/*6 or *6/*6 diplotype.(65)<br>AA + hematopoietic stem cell transplant + cyclophosphamide: decreased, but not absent, risk for oral mucositis vs AG or GG genotype. (64)
*CYP2B6*9*	rs3745274	516G > T (A)(18)	19: 41006936		Missense<br>T: Gln172His<br>T: Gln140His<br>A: Gln172 =<br>A: Gln140 =<br><br>Decreased metabolism	CYP2B6 *6 (G516T and A785G) variant associated with decreased bupropion metabolism.(66)<br><br>GG genotype + Breast Cancer + cyclophosphamide and doxorubicin: likely to require dose reduction vs TT genotype.(26)<br><br>Leukemia patients treated with cyclophosphamide receiving HLA-identical hematopoietic stem cell transplantation from donors with the GG genotype have increased risk of developing veno-occlusive disease of the liver vs donor cells with the GT or TT genotype. (64)<br><br>*1/*1 genotype + chronic lymphocytic leukemia + combination cyclophosphamide and fludarabine: higher chance complete response + higher risk of drug toxicities vs *1/*6 or *6/*6 genotype.(9)<br><br>CYP2B6*1/*1 diplotype + B-cell non-Hodgkin's lymphoma: increased clearance of cyclophosphamide vs *1/*6 or *6/*6 diplotype.(65)
*CYP2B6 *1G* (60)	rs4802101	T>C,A,G<br>-750 T>C (18)	19: 40990556	Regulatory region variant<br>Enhancer?<br>-750 T>C	Decreased metabolism(18)<br>Decreased cyclophosphamide metabolism	CC genotype: decreased metabolism of cyclophosphamide = decreased concentrations of active cyclophosphamide metabolites + decreased risk of gastrointestinal toxicity or leukopenia vs CT or TT genotypes.(18)<br>Chinese population: high LD (∣D′∣ = 0.894) between 64C > T (rs8192709) and -750 T > C (rs4802101).(18)

Figure S1. Tertiles of AMH levels

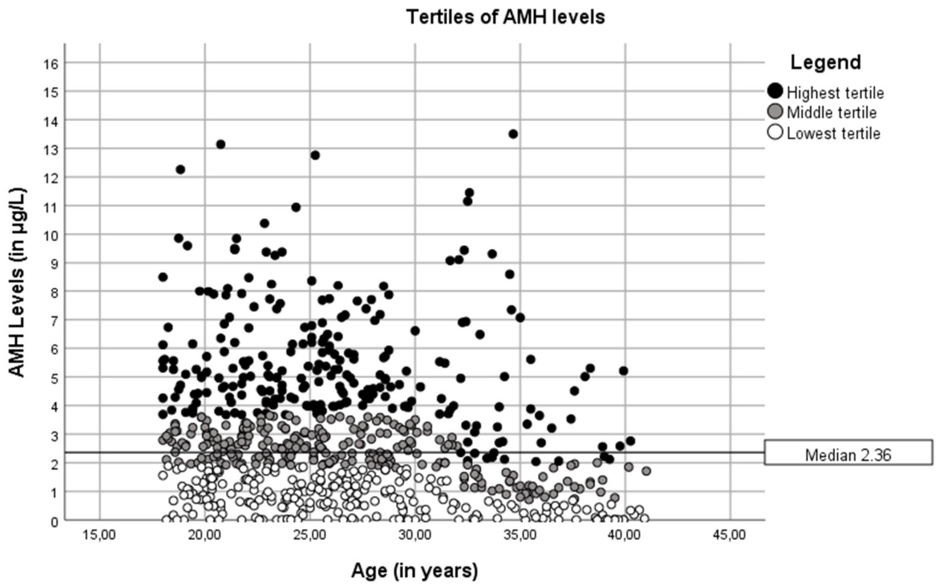

AMH = Anti-Müllerian Hormone

Figure S2. SD scores of LogAMH levels

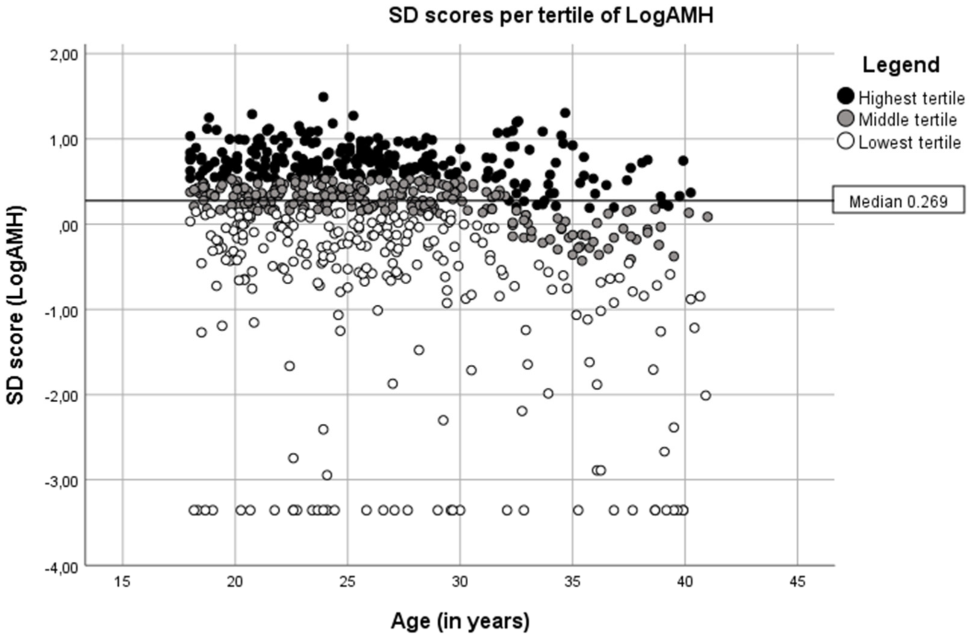

SD = standard deviation; logAMH = logtransformed Anti-Müllerian Hormone

Figure S3. SD scores of LogAMH

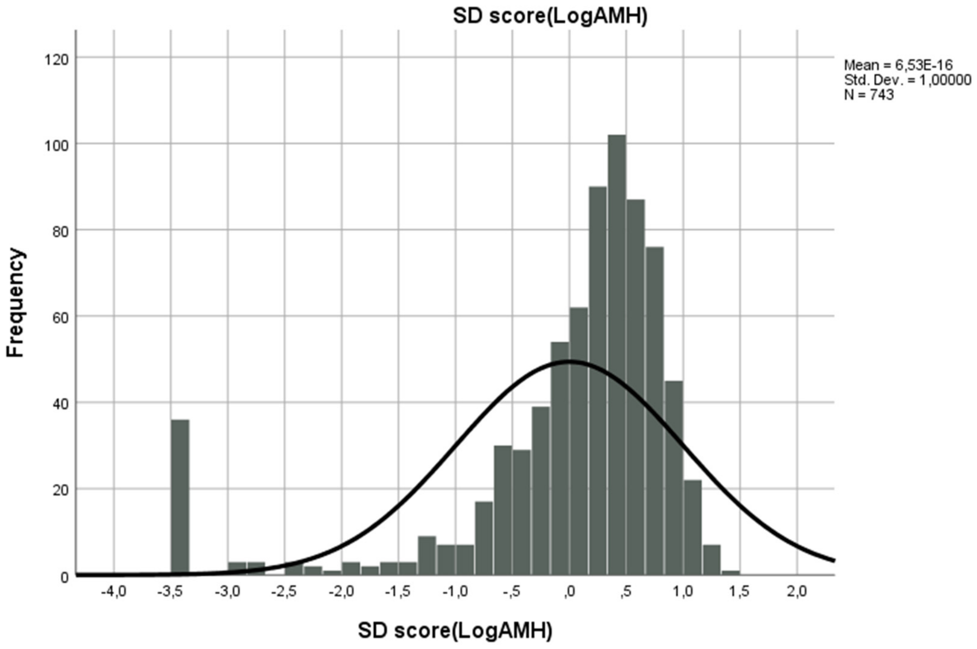

SD = standard deviation; logAMH = logtransformed Anti-Müllerian Hormone

Figure S4. Cyclophosphamide major biotransformation pathways

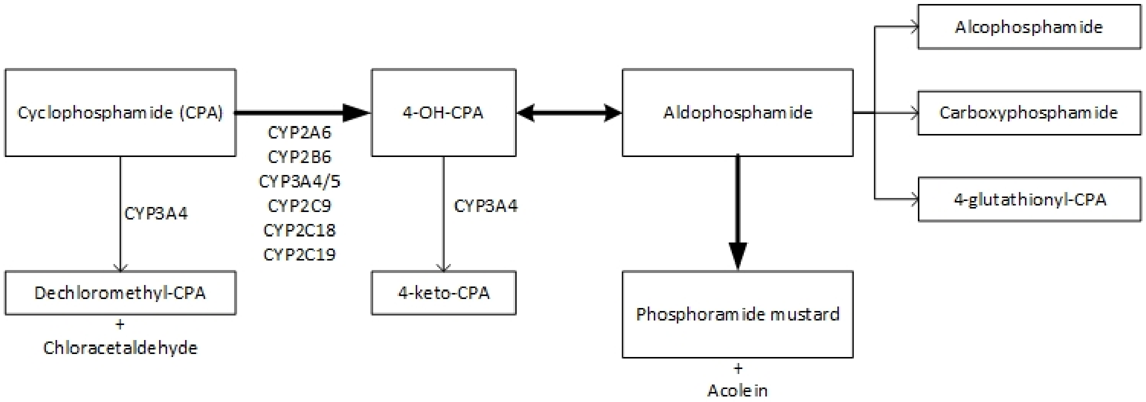

Cyclophosphamide (CPA) is a prodrug whose activation to 4-hydroxycyclophosphamide (4-OH-CPA) is catalyzed by the hepatic cytochrome P450 (CYP) isozymes including CYP2A6, 2B6, 3A4, 3A5, 2C9, 2C18 and 2C19. The highest 4-hydroxylase activity is displayed by CYP2B6. 4-OH-CPA and aldophosphamide are in tautomeric equilibrium. Without enzymatic involvement, aldophosphamide then forms the active phosphoramide mustard and the byproduct acrolein. In alternative pathways, dechloromethyl-CPA and chloroacetaldehyde are formed mediated by CYP3A4/5. Also inactive 4-keto-CPA, carboxyphosphamide, alcophosphamide and 4-glutathionyl-CPA can be formed. (10, 67)

Supplementary references

1. van Dorp W, van den Heuvel-Eibrink MM, Stolk L, Pieters R, Uitterlinden AG, Visser JA, et al. Genetic variation may modify ovarian reserve in female childhood cancer survivors. Hum Reprod. 2013;28(4):1069-76.

2. Anderson CA, Pettersson FH, Clarke GM, Cardon LR, Morris AP, Zondervan KT. Data quality control in genetic case-control association studies. Nat Protoc. 2010;5(9):1564-73.

3. Das S, Forer L, Schonherr S, Sidore C, Locke AE, Kwong A, et al. Next-generation genotype imputation service and methods. Nat Genet. 2016;48(10):1284-7.

4. McCarthy S, Das S, Kretzschmar W, Delaneau O, Wood AR, Teumer A, et al. A reference panel of 64,976 haplotypes for genotype imputation. Nat Genet. 2016;48(10):1279-83.

5. Ikram MA, Brusselle GGO, Murad SD, van Duijn CM, Franco OH, Goedegebure A, et al. The Rotterdam Study: 2018 update on objectives, design and main results. Eur J Epidemiol. 2017;32(9):807-50.

6. Medina-Gomez C, Felix JF, Estrada K, Peters MJ, Herrera L, Kruithof CJ, et al. Challenges in conducting genome-wide association studies in highly admixed multi-ethnic populations: the Generation R Study. Eur J Epidemiol. 2015;30(4):317-30.

7. Brooke RJ, Im C, Wilson CL, Krasin MJ, Liu Q, Li Z, et al. A High-risk Haplotype for Premature Menopause in Childhood Cancer Survivors Exposed to Gonadotoxic Therapy. J Natl Cancer Inst. 2018;110(8):895-904.

8. Ekhart C, Rodenhuis S, Smits PH, Beijnen JH, Huitema AD. Relations between polymorphisms in drug-metabolising enzymes and toxicity of chemotherapy with cyclophosphamide, thiotepa and carboplatin. Pharmacogenet Genomics. 2008;18(11):1009-15.

9. Johnson GG, Lin K, Cox TF, Oates M, Sibson DR, Eccles R, et al. CYP2B6*6 is an independent determinant of inferior response to fludarabine plus cyclophosphamide in chronic lymphocytic leukemia. Blood. 2013;122(26):4253-8.

10. Rodriguez-Antona C, Ingelman-Sundberg M. Cytochrome P450 pharmacogenetics and cancer. Oncogene. 2006;25(11):1679-91.

11. Huezo-Diaz P, Uppugunduri CR, Tyagi AK, Krajinovic M, Ansari M. Pharmacogenetic aspects of drug metabolizing enzymes in busulfan based conditioning prior to allogenic hematopoietic stem cell transplantation in children. Curr Drug Metab. 2014;15(3):251-64.

12. ten Brink MH, Zwaveling J, Swen JJ, Bredius RG, Lankester AC, Guchelaar HJ. Personalized busulfan and treosulfan conditioning for pediatric stem cell transplantation: the role of pharmacogenetics and pharmacokinetics. Drug Discov Today. 2014;19(10):1572-86.

13. Kuhne A, Kaiser R, Schirmer M, Heider U, Muhlke S, Niere W, et al. Genetic polymorphisms in the amino acid transporters LAT1 and LAT2 in relation to the pharmacokinetics and side effects of melphalan. Pharmacogenet Genomics. 2007;17(7):505-17.

14. Pandya U, Srivastava SK, Singhal SS, Pal A, Awasthi S, Zimniak P, et al. Activity of allelic variants of Pi class human glutathione S-transferase toward chlorambucil. Biochem Biophys Res Commun. 2000;278(1):258-62.

15. Yates AD, Achuthan P, Akanni W, Allen J, Allen J, Alvarez-Jarreta J, et al. Ensembl 2020. Nucleic Acids Res. 2020;48(D1):D682-D8.

16. van Schaik RH. Cancer treatment and pharmacogenetics of cytochrome P450 enzymes. Invest New Drugs. 2005;23(6):513-22.

17. van Schaik RH. CYP450 pharmacogenetics for personalizing cancer therapy. Drug Resist Updat. 2008;11(3):77-98.

18. Shu WY, Guan S, Yang XY, Liang LQ, Li JL, Chen ZJ, et al. Genetic markers in CYP2C19 and CYP2B6 for prediction of cyclophosphamide's 4-hydroxylation, efficacy and side effects in Chinese patients with systemic lupus erythematosus. Brit J Clin Pharmaco. 2016;81(2):327-40.

19. Bertholee D, Maring JG, van Kuilenburg AB. Genotypes Affecting the Pharmacokinetics of Anticancer Drugs. Clin Pharmacokinet. 2017;56(4):317-37.

20. Clinical Pharmacogenetics Implementation Consortium (St. Jude Children's Research Hospital and Stanford University) C. ClinVar CYP2C19. 2018.
21. Landrum MJ, Lee JM, Benson M, Brown GR, Chao C, Chitipiralla S, et al. ClinVar: improving access to variant interpretations and supporting evidence. Nucleic Acids Res. 2018;46(D1):D1062-D7.
22. Rudberg I, Mohebi B, Hermann M, Refsum H, Molden E. Impact of the ultrarapid CYP2C19*17 allele on serum concentration of escitalopram in psychiatric patients. Clin Pharmacol Ther. 2008;83(2):322-7.
23. Nakamoto K, Kidd JR, Jenison RD, Klaassen CD, Wan YJ, Kidd KK, et al. Genotyping and haplotyping of CYP2C19 functional alleles on thin-film biosensor chips. Pharmacogenet Genomics. 2007;17(2):103-14.
24. Whirl-Carrillo M, McDonagh EM, Hebert JM, Gong L, Sangkuhl K, Thorn CF, et al. Pharmacogenomics knowledge for personalized medicine. Clin Pharmacol Ther. 2012;92(4):414-7.
25. Tecza K, Pamula-Pilat J, Lanuszewska J, Butkiewicz D, Grzybowska E. Pharmacogenetics of toxicity of 5-fluorouracil, doxorubicin and cyclophosphamide chemotherapy in breast cancer patients. Oncotarget. 2018;9(10):9114-36.
26. Bray J, Sludden J, Griffin MJ, Cole M, Verrill M, Jamieson D, et al. Influence of pharmacogenetics on response and toxicity in breast cancer patients treated with doxorubicin and cyclophosphamide. Br J Cancer. 2010;102(6):1003-9.
27. Singh G, Saxena N, Aggarwal A, Misra R. Cytochrome P450 polymorphism as a predictor of ovarian toxicity to pulse cyclophosphamide in systemic lupus erythematosus. J Rheumatol. 2007;34(4):731-3.
28. Takada K, Arefayene M, Desta Z, Yarboro CH, Boumpas DT, Balow JE, et al. Cytochrome P450 pharmacogenetics as a predictor of toxicity and clinical response to pulse cyclophosphamide in lupus nephritis. Arthritis Rheum. 2004;50(7):2202-10.
29. Timm R, Kaiser R, Lotsch J, Heider U, Sezer O, Weisz K, et al. Association of cyclophosphamide pharmacokinetics to polymorphic cytochrome P450 2C19. Pharmacogenomics J. 2005;5(6):365-73.
30. Sim SC, Risinger C, Dahl ML, Aklillu E, Christensen M, Bertilsson L, et al. A common novel CYP2C19 gene variant causes ultrarapid drug metabolism relevant for the drug response to proton pump inhibitors and antidepressants. Clin Pharmacol Ther. 2006;79(1):103-13.
31. Pharmvar. Pharmvar Cyp3A4.
32. Sherry ST, Ward MH, Kholodov M, Baker J, Phan L, Smigielski EM, et al. dbSNP: the NCBI database of genetic variation. Nucleic Acids Res. 2001;29(1):308-11.
33. Su HI, Sammel MD, Velders L, Horn M, Stankiewicz C, Matro J, et al. Association of cyclophosphamide drug-metabolizing enzyme polymorphisms and chemotherapy-related ovarian failure in breast cancer survivors. Fertil Steril. 2010;94(2):645-54.
34. Hesselink DA, van Schaik RH, van der Heiden IP, van der Werf M, Gregoor PJ, Lindemans J, et al. Genetic polymorphisms of the CYP3A4, CYP3A5, and MDR-1 genes and pharmacokinetics of the calcineurin inhibitors cyclosporine and tacrolimus. Clin Pharmacol Ther. 2003;74(3):245-54.
35. Amirimani B, Walker AH, Weber BL, Rebbeck TR. RESPONSE: re: modification of clinical presentation of prostate tumors by a novel genetic variant in CYP3A4. J Natl Cancer Inst. 1999;91(18):1588-90.
36. Grimsrud KN, Ivanova X, Sherwin CM, Palmieri TL, Tran NK. Identification of Cytochrome P450 Polymorphisms in Burn Patients and Impact on Fentanyl Pharmacokinetics: A Pilot Study. J Burn Care Res. 2019;40(1):91-6.
37. Wishart DS, Feunang YD, Guo AC, Lo EJ, Marcu A, Grant JR, et al. DrugBank 5.0: a major update to the DrugBank database for 2018. Nucleic Acids Res. 2018;46(D1):D1074-D82.
38. Amirimani B, Ning B, Deitz AC, Weber BL, Kadlubar FF, Rebbeck TR. Increased transcriptional activity of the CYP3A4*1B promoter variant. Environ Mol Mutagen. 2003;42(4):299-305.
39. Floyd MD, Gervasini G, Masica AL, Mayo G, George AL, Jr., Bhat K, et al. Genotype-phenotype associations for common CYP3A4 and CYP3A5 variants in the basal and induced metabolism of

midazolam in European- and African-American men and women. Pharmacogenetics. 2003;13(10):595-606.
40. Gervasini G, Garcia M, Macias RM, Cubero JJ, Caravaca F, Benitez J. Impact of genetic polymorphisms on tacrolimus pharmacokinetics and the clinical outcome of renal transplantation. Transpl Int. 2012;25(4):471-80.
41. Kuehl P, Zhang J, Lin Y, Lamba J, Assem M, Schuetz J, et al. Sequence diversity in CYP3A promoters and characterization of the genetic basis of polymorphic CYP3A5 expression. Nat Genet. 2001;27(4):383-91.
42. Miao J, Jin Y, Marunde RL, Gorski CJ, Kim S, Quinney S, et al. Association of genotypes of the CYP3A cluster with midazolam disposition in vivo. Pharmacogenomics J. 2009;9(5):319-26.
43. Sinues B, Vicente J, Fanlo A, Vasquez P, Medina JC, Mayayo E, et al. CYP3A5*3 and CYP3A4*1B allele distribution and genotype combinations: differences between Spaniards and Central Americans. Ther Drug Monit. 2007;29(4):412-6.
44. Lamba JK, Lin YS, Thummel K, Daly A, Watkins PB, Strom S, et al. Common allelic variants of cytochrome P4503A4 and their prevalence in different populations. Pharmacogenetics. 2002;12(2):121-32.
45. Spurdle AB, Goodwin B, Hodgson E, Hopper JL, Chen X, Purdie DM, et al. The CYP3A4*1B polymorphism has no functional significance and is not associated with risk of breast or ovarian cancer. Pharmacogenetics. 2002;12(5):355-66.
46. DeMichele A, Gimotty P, Botbyl J, Aplenc R, Colligon T, Foulkes AS, et al. In response to "Drug metabolizing enzyme polymorphisms predict clinical outcome in a node-positive breast cancer cohort". J Clin Oncol. 2007;25(35):5675-7.
47. Paris PL, Kupelian PA, Hall JM, Williams TL, Levin H, Klein EA, et al. Association between a CYP3A4 genetic variant and clinical presentation in African-American prostate cancer patients. Cancer Epidemiol Biomarkers Prev. 1999;8(10):901-5.
48. Rebbeck TR, Jaffe JM, Walker AH, Wein AJ, Malkowicz SB. Modification of clinical presentation of prostate tumors by a novel genetic variant in CYP3A4. J Natl Cancer Inst. 1998;90(16):1225-9.
49. Ekhart C, Doodeman VD, Rodenhuis S, Smits PH, Beijnen JH, Huitema AD. Influence of polymorphisms of drug metabolizing enzymes (CYP2B6, CYP2C9, CYP2C19, CYP3A4, CYP3A5, GSTA1, GSTP1, ALDH1A1 and ALDH3A1) on the pharmacokinetics of cyclophosphamide and 4-hydroxycyclophosphamide. Pharmacogenet Genomics. 2008;18(6):515-23.
50. Xie H, Griskevicius L, Stahle L, Hassan Z, Yasar U, Rane A, et al. Pharmacogenetics of cyclophosphamide in patients with hematological malignancies. Eur J Pharm Sci. 2006;27(1):54-61.
51. Sata F, Sapone A, Elizondo G, Stocker P, Miller VP, Zheng W, et al. CYP3A4 allelic variants with amino acid substitutions in exons 7 and 12: evidence for an allelic variant with altered catalytic activity. Clin Pharmacol Ther. 2000;67(1):48-56.
52. van Schaik RH, de Wildt SN, Brosens R, van Fessem M, van den Anker JN, Lindemans J. The CYP3A4*3 allele: is it really rare? Clin Chem. 2001;47(6):1104-6.
53. Thompson JF, Man M, Johnson KJ, Wood LS, Lira ME, Lloyd DB, et al. An association study of 43 SNPs in 16 candidate genes with atorvastatin response. Pharmacogenomics J. 2005;5(6):352-8.
54. Saiz-Rodriguez M, Almenara S, Navares-Gomez M, Ochoa D, Roman M, Zubiaur P, et al. Effect of the Most Relevant CYP3A4 and CYP3A5 Polymorphisms on the Pharmacokinetic Parameters of 10 CYP3A Substrates. Biomedicines. 2020;8(4).
55. Kitzmiller JP, Luzum JA, Dauki A, Krauss RM, Medina MW. Candidate-Gene Study of Functional Polymorphisms in SLCO1B1 and CYP3A4/5 and the Cholesterol-Lowering Response to Simvastatin. Clin Transl Sci. 2017;10(3):172-7.
56. Diekstra MH, Liu X, Swen JJ, Boven E, Castellano D, Gelderblom H, et al. Association of single nucleotide polymorphisms in IL8 and IL13 with sunitinib-induced toxicity in patients with metastatic renal cell carcinoma. Eur J Clin Pharmacol. 2015;71(12):1477-84.

57. Elens L, Nieuweboer A, Clarke SJ, Charles KA, de Graan AJ, Haufroid V, et al. CYP3A4 intron 6 C>T SNP (CYP3A4*22) encodes lower CYP3A4 activity in cancer patients, as measured with probes midazolam and erythromycin. Pharmacogenomics. 2013;14(2):137-49.
58. de Graan AJ, Elens L, Sprowl JA, Sparreboom A, Friberg LE, van der Holt B, et al. CYP3A4*22 genotype and systemic exposure affect paclitaxel-induced neurotoxicity. Clin Cancer Res. 2013;19(12):3316-24.
59. Kitzmiller JP, Mikulik EB, Dauki AM, Murkherjee C, Luzum JA. Pharmacogenomics of statins: understanding susceptibility to adverse effects. Pharmgenomics Pers Med. 2016;9:97-106.
60. (PharmVar) PVC. (Gaedigk et al. 2018, CPT 103:399; Gaedigk et al. 2019, CPT 105:29); [Available from: www.PharmVar.org.
61. Nakajima M, Komagata S, Fujiki Y, Kanada Y, Ebi H, Itoh K, et al. Genetic polymorphisms of CYP2B6 affect the pharmacokinetics/pharmacodynamics of cyclophosphamide in Japanese cancer patients. Pharmacogenet Genomics. 2007;17(6):431-45.
62. Xie HJ, Yasar U, Lundgren S, Griskevicius L, Terelius Y, Hassan M, et al. Role of polymorphic human CYP2B6 in cyclophosphamide bioactivation. Pharmacogenomics J. 2003;3(1):53-61.
63. Aminkeng F, Ross CJ, Rassekh SR, Brunham LR, Sistonen J, Dube MP, et al. Higher frequency of genetic variants conferring increased risk for ADRs for commonly used drugs treating cancer, AIDS and tuberculosis in persons of African descent. Pharmacogenomics J. 2014;14(2):160-70.
64. Rocha V, Porcher R, Fernandes JF, Filion A, Bittencourt H, Silva W, Jr., et al. Association of drug metabolism gene polymorphisms with toxicities, graft-versus-host disease and survival after HLA-identical sibling hematopoietic stem cell transplantation for patients with leukemia. Leukemia. 2009;23(3):545-56.
65. Veal GJ, Cole M, Chinnaswamy G, Sludden J, Jamieson D, Errington J, et al. Cyclophosphamide pharmacokinetics and pharmacogenetics in children with B-cell non-Hodgkin's lymphoma. Eur J Cancer. 2016;55:56-64.
66. Ray R, Tyndale RF, Lerman C. Nicotine dependence pharmacogenetics: role of genetic variation in nicotine-metabolizing enzymes. J Neurogenet. 2009;23(3):252-61.
67. Roy P, Yu LJ, Crespi CL, Waxman DJ. Development of a substrate-activity based approach to identify the major human liver P-450 catalysts of cyclophosphamide and ifosfamide activation based on cDNA-expressed activities and liver microsomal P-450 profiles. Drug Metab Dispos. 1999;27(6):655-66.
